# Inflammatory Stimuli Reprogram Macrophage Phagocytosis to Macropinocytosis for the Rapid Elimination of Pathogens

**DOI:** 10.1371/journal.ppat.1003879

**Published:** 2014-01-30

**Authors:** Somdeb BoseDasgupta, Jean Pieters

**Affiliations:** Biozentrum, University of Basel, Basel, Switzerland; Weill Medical College of Cornell University, United States of America

## Abstract

Following an infectious challenge, macrophages have to be activated in order to allow efficient clearance of infectious pathogens, but how macrophage activation is coupled to increased clearance remains largely unknown. We here describe that inflammatory stimuli induced the reprogramming of the macrophage endocytic machinery from receptor-mediated phagocytosis to macropinocytosis, allowing the rapid transfer of internalized cargo to lysosomes in a receptor-independent manner. Reprogramming occurred through protein kinase C-mediated phosphorylation of the macrophage protein coronin 1, thereby activating phosphoinositol (PI)-3-kinase activity necessary for macropinocytic uptake. Expression of a phosphomimetic form of coronin 1 was sufficient to induce PI3-kinase activation and macropinocytosis even in the absence of inflammatory stimuli. Together these results suggest a hitherto unknown mechanism to regulate the internalization and degradation of infectious material during inflammation.

## Author Summary

The main cells that are involved in cleaning up microbial pathogens are macrophages. Upon an infection, macrophages are being recruited to the site of infection by a number of different stimuli. In addition, during an infection, macrophages are also activated by cytokines such as interferon-γ and tumor necrosis factor-α that is released from other immune cells. Such macrophage activation is important to achieve a rapid and efficient clearance of microbial pathogens. In this study, we found that macrophage activation induces uptake through macropinocytosis rather than receptor-mediate phagocytosis. As a consequence, microbial material as well as particles can be internalized far more efficiently; In addition, the internalized cargo is rapidly destroyed within lysosomes. We also provide the mechanisms for the switch from phagocytosis to macropinocytosis, which turned out to be the cytokine-induced phosphorylation of the host protein coronin 1. Phosphorylated coronin 1 activated the lipid kinase phosphoinositide 3-kinase, which is known to be responsible for the entry of cargo through macropinocytosis. Together these results provide evidence for a hitherto unrecognized mechanisms to regulate the internalization and degradation of infectious material during an infection.

## Introduction

Macrophages are the main scavengers responsible for clearance of solutes and particulate material as well as to act as defense cells against invading microbes [1]. The main mechanisms via which macrophages can internalize and clear microbial material occurs through receptor-mediated phagocytosis. This process, making use of different cell surface receptors, including Fc receptors, complement receptors, scavenging receptors as well as several lectin receptors, ensure the uptake of particulate material into phagosomes followed by delivery of the cargo to lysosomes [1].

Under certain conditions phagocytosis is not sufficient for an effective elimination of microbial pathogens. For example, pathogenic mycobacteria, which include the causative agent of tuberculosis, can be internalized via phagocytosis using different receptors, including complement receptor, scavenging receptors as well as lectin receptors such as the mannose receptor and DC-SIGN [2] [1]. Once internalized into phagosomes, pathogenic mycobacteria have evolved to withstand lysosomal degradation by effectively blocking phagosome-lysosome fusion thereby surviving within macrophage phagosomes instead of being degraded in lysosomes prior to cytosolic escape [3]–[10]. Also, during an acute infection, the phagocytic capacity of macrophages may become limiting in being able to internalize and destroy sufficient numbers of bacilli in order to curb the infection [11]. Furthermore, the particular receptor involved may modulate the macrophage killing capacity by silencing certain macrophage responses such as the respiratory burst [12].

As an alternative to receptor-mediated phagocytosis, macrophages can also internalize material via macropinocytosis, a non-saturable mode of uptake that allows the internalization of large amounts of cargo independent of any receptor usage [11], [13]–[15]. In several cell types, macropinocytosis can be transiently induced by growth factors as well as certain pathogens such as *Salmonella*, *Shigella* or viruses [16], [17]. In macrophages, as well as dendritic cells, where macropinocytosis also occurs constitutively, macropinocytosis allows to efficiently process infectious material as well as activate immune responses [14].

Here, we show that inflammatory stimuli reprogram the macrophage endocytic pathway from receptor-mediated phagocytosis to macropinocytosis, enabling macrophages to internalize large amounts of cargo for direct transfer to lysosomes. We found that upon macrophage activation, serine phosphorylation of the macrophage protein coronin 1 is the key molecular switch that reprograms the macrophage from a phagocytic uptake mode to macropinocytosis. Coronin 1 (also known as P57 or TACO, for Tryptophan aspartate containing Coat protein), was originally identified as a survival factor for intracellular residing mycobacteria by blocking the delivery of pathogenic mycobacteria to lysosomes via the activation of the Ca^2+^/calcineurin pathway [18]–[23]. In resting, non-activated macrophages, coronin 1 is associated with the cell cortex via an interaction (either direct or indirect) with plasma membrane cholesterol [24]. We found that upon cytokine-mediated macrophage activation, coronin 1 was phosphorylated on multiple serine residues by protein kinase C, which induced the relocation of coronin 1 from the cortex to cytoplasmic puncta. Serine phosphorylation of coronin 1 was sufficient to induce phosphoinositol-3-kinase activity thereby switching the internalization mode from receptor-mediated phagocytosis to macropinocytosis. Together these results not only provide a molecular explanation for the mycobactericidal effect of macrophage activating cytokines, but furthermore suggest that macrophage activation reprograms the endocytic machinery through coronin 1 phosphorylation in order to efficiently eliminate infectious cargo.

## Results

### Mycobacterial Entry in Activated Macrophages Occurs through Macropinocytosis

**Figure 1:**
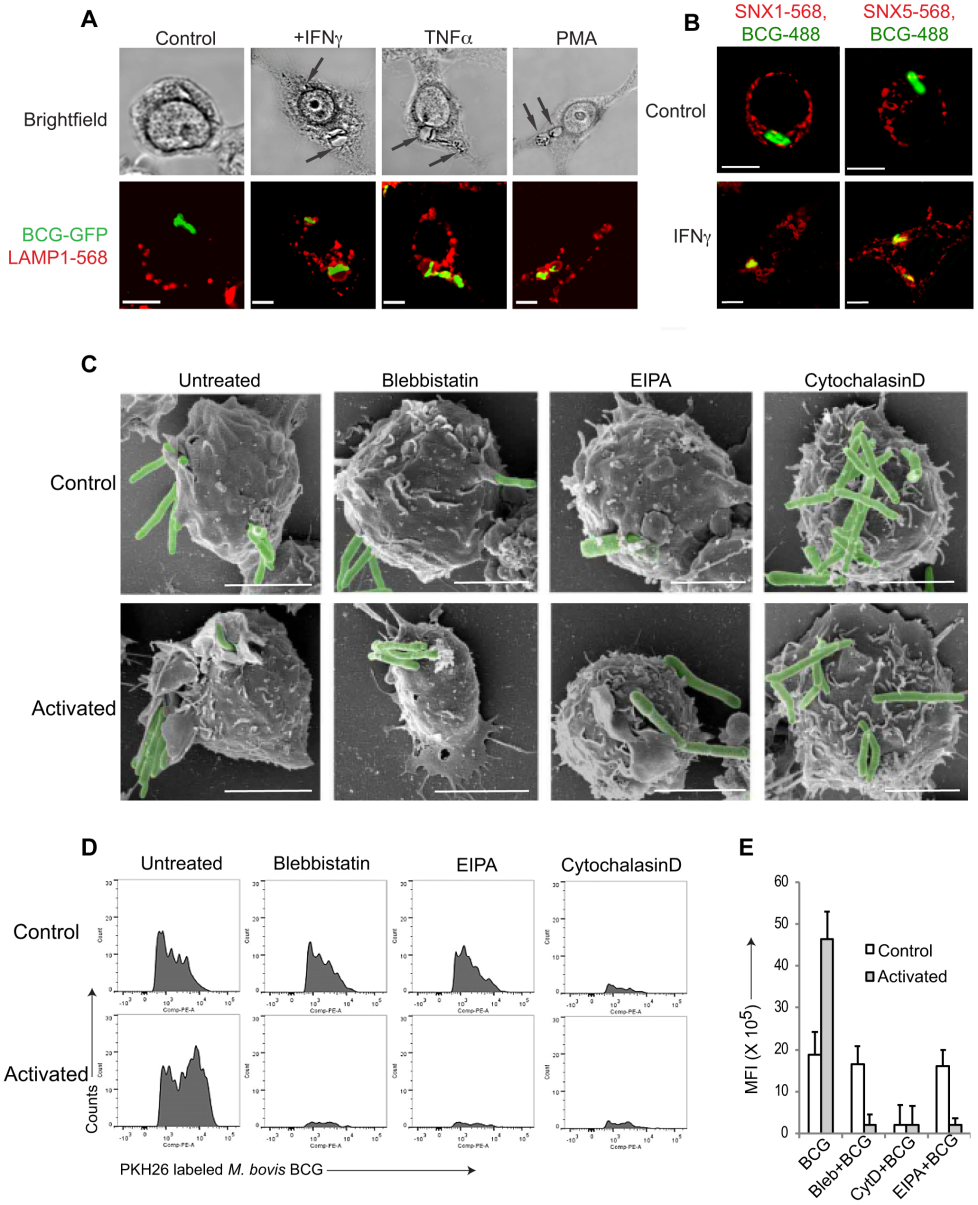
Macrophage activation results in mycobacterial entry via macropinocytosis. A. Macrophages were left untreated or activated with IFN-γ (1000 U/ml), TNFα (1000 U/ml) for 20 hr or PMA (100 nM) for 4 hrs followed by infection with *M. bovis* BCG-GFP (green) for 3 hours. Cells were fixed and incubated with anti-LAMP1 antibodies followed by AlexaFluor568-conjugated secondary antibodies. Bar: 10 µm. B. Macrophages were left untreated or activated with IFN-γ (1000 U/ml) for 20 hrs followed by infection with *M. bovis* BCG-GFP (green) for 30 min, fixed and incubated with anti-SNX1 and anti-SNX5 antibodies followed by incubation using AlexaFluor568-conjugated secondary antibodies. Bar:10 µm. C. Scanning electron micrographs showing the entry of *M. bovis* BCG-GFP, in resting or IFN-γ-activated (20 hrs) macrophages in the absence or presence of blebbistatin (150 µM), amiloride (EIPA, 50 µM), or cytochalasin D (10 µg/ml) respectively upon incubation with mycobacteria for 90 min., Bar: 200 nm. D. Resting (top panel) or IFN-γ-activated (20 hrs) (bottom panel) macrophages were incubated with PKH26 labeled *M. bovis BCG* in the absence or presence of blebbistatin, EIPA or Cytochalasin D for 60 min. followed by flow cytometry analysis. For each condition, 10'000 cells were analyzed. Shown is a representative profile out of 3 independent experiments. E. The mean fluorescence intensity of PKH26 labeled *M. bovis BCG* uptake in resting versus interferon-γ-activated macrophages obtained for 10,000 cells (samples were analyzed in duplicate from 3 independent experiments and plotted).

Macrophage activation by either interferon-γ (IFN-γ) or tumor necrosis factor-α results in the rapid delivery of the internalized mycobacteria to lysosomes followed by mycobacterial killing (see Fig. 1A, Fig. S1A,B and [25]). Interestingly, close inspection of the mycobacterial internalization process in activated macrophages by light microscopy showed that mycobacteria entered macrophages in large spacious vacuoles (Fig. 1A, arrows), which is an indication that the bacilli entered cells via macropinocytosis, rather than phagocytosis [14], [26]–[29]. Upon addition of the phorbol ester phorbol 12-myristate 13-acetate PMA), that is known to induce macropinocytosis [30], [31], similar spacious vacuoles were observed (Fig. 1A).

To further analyze entry of mycobacteria in activated macrophages, resting, i.e., non-activated, or activated macrophages were infected with mycobacteria, fixed, and analyzed by immunofluorescence microscopy. Consistent with macropinocytic uptake, both early macropinosomal markers sorting nexin 1 and sorting nexin 5 [13] strongly colocalized with mycobacterial vacuoles in activated, but not in resting macrophages (Fig. 1B and Fig. S1C–E). Furthermore, internalization of GFP-expressing mycobacteria in IFN-γ-activated macrophages was prevented by the macropinocytic inhibitors amiloride and 3-methyladenine [32], [33] only in activated macrophages (Fig. S1F) as judged by the presence of GFP immunoreactivity in macrophage lysates following mycobacterial uptake. In contrast, the actin depolymerizing agent cytochalasin D prevented bacterial entry in both resting as well as activated macrophages (Fig. S1F). An inhibitor of clathrin-mediated endocytosis, monodansyl cadaverine (MDC; [34]) on the other hand could not inhibit internalization of mycobacteria in resting or activated macrophages (Fig. S1F).

Macropinocytic entry is associated with the formation of large membrane ruffles and blebs at the site of uptake [14], [26], [35]. To qualitatively assess phagocytosis versus macropinocytosis, scanning electron microscopy was used. As shown in Fig. 1C, when macrophages that had either been untreated or activated by interferon-γ were allowed to internalize mycobacteria, this resulted in the presence of extensive membrane ruffles in activated, but not resting macrophages, characteristic of macropinocytic uptake (Fig. 1C). Similarly, macrophages infected in the presence of the macropinocytosis inhibitors blebbistatin [36] or 5-(N-Ethyl-N-isopropyl) amiloride (EIPA) exhibited no membrane ruffles, and macropinocytic entry was not observed. Treatment of cells with the cytoskeletal inhibitor cytochalasin D prevented mycobacterial entry in both resting and activated cells (Fig. 1C).

To further analyze entry through phagocytosis versus macropinocytosis, we established a fluorescence activated cell sorting (FACS) assay, that allowed to distinguish phagocytosis from macropinocytosis in a quantitative manner. To that end, macrophages were incubated either with IgG-coated AlexaFluor568-conjugated beads (to asses phagocytosis) or rhodamine-conjugated dextran 70000 (to asses macropinocytosis) and analyzed by flow cytometry. Internalization of IgG-coated beads resulted in the appearance of several peaks, with the highest peak corresponding to cells having internalized a single bead, with a gradual decrease in the number of cells internalizing multiple beads which is consistent with phagocytic uptake (Fig. S1G, left panel). In contrast, internalization of rhodamine-coupled dextran resulted in a broad peak of high fluorescence, consistent with macropinocytic uptake (Fig. S1H, left panel). While cytochalasin D blocked bead internalization as well as dextran entry (Fig. S1G,H, right panels), the macropinocytosis inhibitor blebbistatin specifically prevented dextran uptake, while not affecting the internalization of IgG-coated beads (Fig. S1G,H middle panels).

Using this assay, we analyzed the uptake of mycobacteria in both resting as well as activated macrophages. As shown in Fig. 1D, incubation of non activated macrophages with fluorescently labeled mycobacteria resulted in a FACS profile reminiscent of phagocytosis, while the incubation of interferon-γ-activated macrophages with mycobacteria revealed a broad peak of fluorescence, consistent with macropinocytic uptake. Moreover, while the actin poisoning agent cytochalasin D blocked uptake of mycobacteria in both resting and activated macrophages, the macropinocytosis inhibitors blebbistatin and EIPA selectively blocked uptake in activated, but not resting macrophages (Fig. 1D,E). Together these results suggest that upon macrophage activation with interferon-γ mycobacteria are engulfed by macropinocytosis instead of phagocytosis.

### Macrophage Activation Reprograms the Endocytic Pathway from Phagocytosis to Macropinocytosis

To analyze whether the switch from phagocytosis to macropinocytosis upon macrophage activation is specific for mycobacterial uptake or represents a general mechanism, we analyzed entry of both *E. coli* as well as *M. marinum* by scanning electron microscopy as well as the aforementioned FACS-based assay. To that end, bacilli were labeled with the fluorescent dye PKH-26. The analysis of entry of *M. marinum* as well as *E. coli* as shown in Fig. S2 shows that the bacteria were internalized into activated macrophages through macropinocytosis as judged by the ability of both blebbistatin as well as EIPA to prevent bacterial entry into activated, but not resting macrophages (Fig. S2).

**Figure 2:**
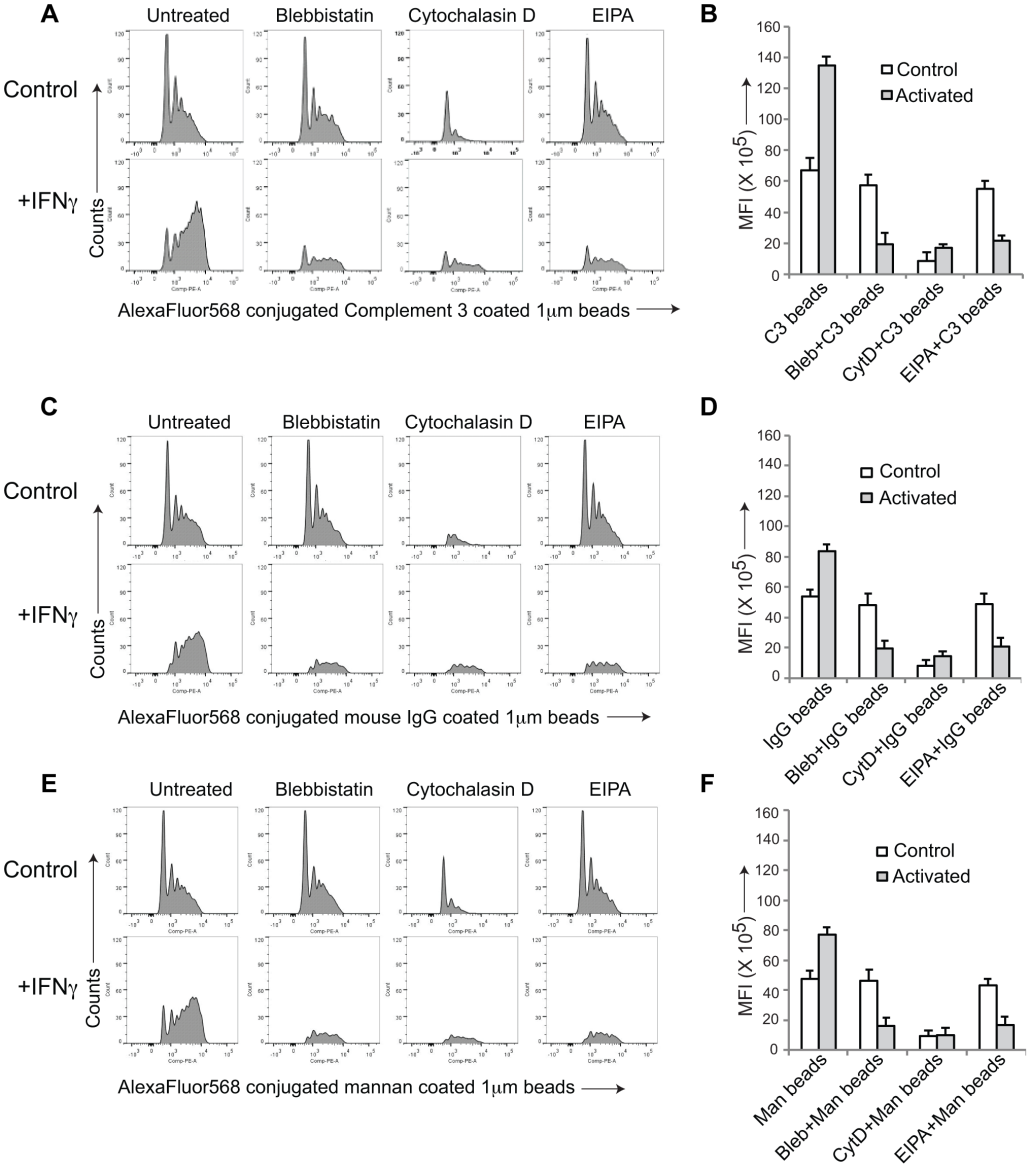
General induction of upon macrophage activation. AlexaFluor 568 conjugated Complement type 3 coated (A,B), mouse IgG coated (C,D) or mannan-coated 1 µM beads (E,F) were added to resting or activated macrophages in the absence or presence of the indicated inhibitors and analyzed by FACS as described in the legend to Figure S1. Panel B, D and F, depict the mean fluorescence intensity in resting versus activated macrophages obtained for 10,000 cells (data represents average of duplicate samples from three independent experiments).

To further asses the capacity of activated macrophages to internalize material via macropinocytosis, we analyzed uptake of fluorescently-labeled beads coated with either complement 3 (Fig. 2 A,B), IgG (Fig. 2 C,D) or mannan (Fig. 2 E,F), that in resting macrophages is internalized through complement type 3, Fc gamma or mannose receptors, respectively. While all cargo entered resting, non-activated macrophages through phagocytosis, upon macrophage activation by interferon-γ, entry occurred through macropinocytosis as judged by the FACS profiles (Fig. 2A,C,E). Furthermore, while in all cases incubation with cytochalasin D prevented entry in both resting and activated macrophages, the macropinocytosis inhibitors blebbistatin and EIPA, did not affect entry into resting macrophages, but blocked the internalization process in activated macrophages. These results therefore strongly suggest that macrophage activation causes a general reprogramming of the entry machinery from phagocytosis to macropinocytosis.

### Macrophage Activation Induces the Relocation of Coronin 1 from the Cell Cortex to Cytoplasmic Puncta in a Protein Kinase C-dependent Manner

**Figure 3:**
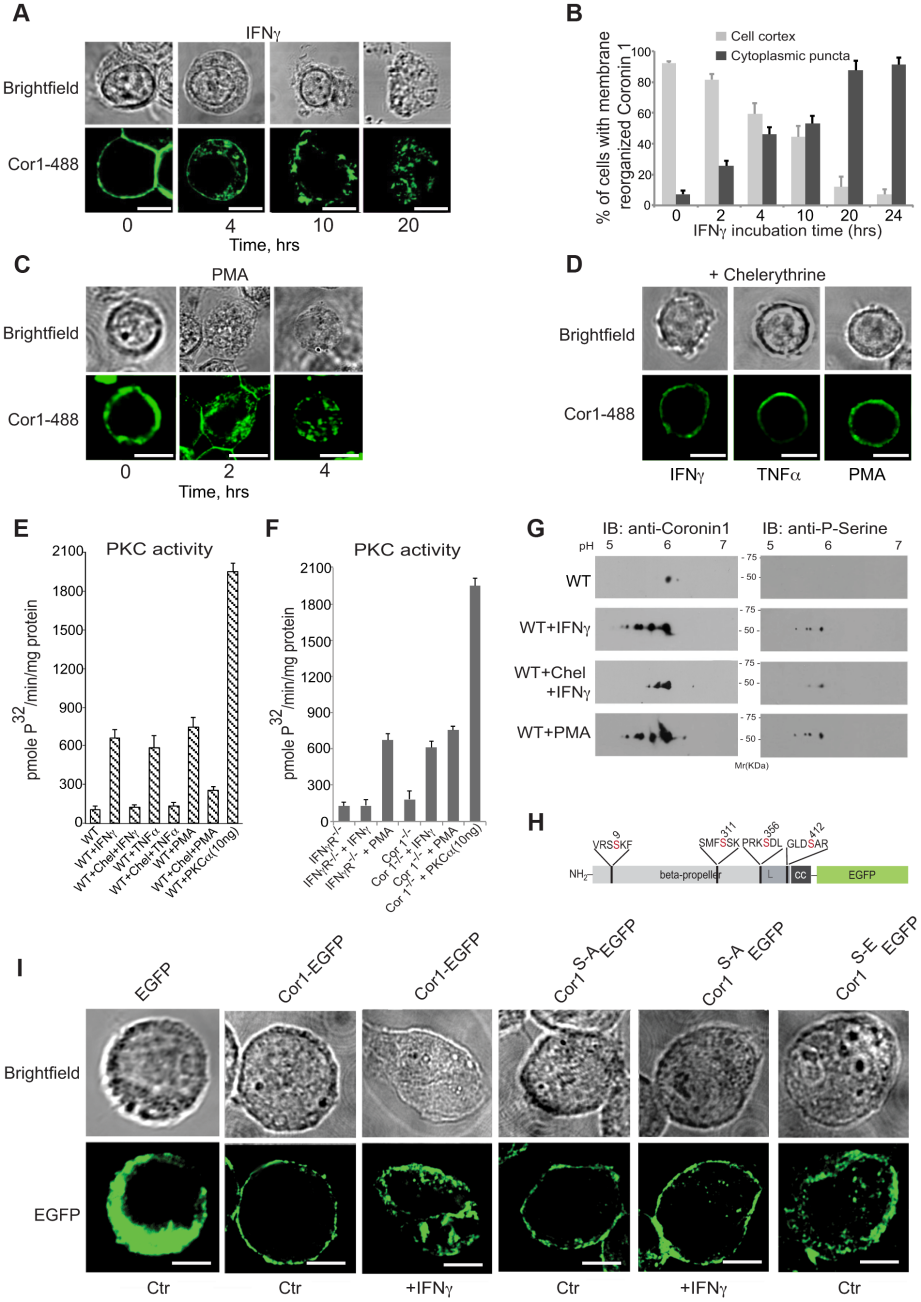
Coronin 1 relocalization and phosphorylation upon macrophage activation. A,B. Macrophages were treated with IFN-γ for the indicated times, followed by fixation and incubation with anti-coronin 1 antibodies followed by AlexaFluor488-conjugated secondary antibodies. Bar:10 µm. In B, the percentage of cortical (light gray) and cytoplasmic punctate (dark gray) localized coronin 1 was quantitated (n = 25 cells; average of triplicate experiments +/−SD). C. Macrophages were treated with PMA for the indicated times, fixed and stained with anti-coronin 1 antibodies followed by AlexaFluor488-conjugated secondary antibodies. Bar:10 µm. D. Macrophages were treated with IFN-γ or TNFα for 20 hrs or PMA for 4 hrs in the presence of chelerythrine, fixed and stained with anti-coronin 1 antibodies followed by AlexaFluor488-conjugated secondary antibodies. Bar:10 µm. E,F. PKC activity in resting versus activated (IFN-γ, TNFα and PMA) wild type macrophages in the absence and presence of chelerythrine (10 µM) (E) or in macrophages from IFN-γR^−/−^ or coronin 1-deficient mice (F). Shown are average values (+/− SD) from three experiments. G. Two-dimensional IEF/SDS-PAGE analysis of purified coronin 1 from resting, IFN-γ-activated macrophages in the absence or presence of chelerythrine or PMA-activated macrophages. Following electrophoresis, the gels were immunoblotted using the antibodies indicated. H. Schematic representation of the coronin 1-EGFP constructs showing the position of the mutated serines. I. Coronin 1-deficient macrophages were transfected with the indicated plasmids and either left untreated or stimulated as indicated and observed by confocal laser scanning microscopy. Bar: 10 µm.

In the course of analyzing IFN-γ-mediated macropinocytic uptake, we noticed the relocation of the macrophage protein coronin 1 [19], [21], [23] from the cell cortex to cytoplasmic puncta (Fig. 3A,B and the Movies S1 and S2). Similarly, both tumor necrosis factor (TNF) α, as well as phorbol 12-myristate 13-acetate, (PMA) a direct activator of macropinocytosis [30], [31] caused coronin 1 delocalization from the cell cortex to the cytoplasm (Fig. 3C and Fig. S3A as well as Movie S3). The coronin 1-containing cytoplasmic puncta were positive for the cholesterol labeling dye filipin as well as for the endocytic vesicle maker FM4-64 (Fig S3B,C). Since PMA is a direct activator of protein kinase C (PKC) [37], we analyzed whether coronin 1 delocalization resulted from cytokine-mediated PKC activation [38]. Indeed, preincubation of macrophages with the PKC inhibitor chelerythrine prior to activation prevented coronin 1 relocalization (Fig. 3D). Furthermore, IFN-γ and tumor necrosis factor-α as well as PMA stimulation resulted in the activation of PKC (Fig. 3E). PKC activation was a direct result of IFN-γ triggering, since stimulation of macrophages isolated from IFN-γ receptor–deficient mice failed to result in PKC activation (Fig. 3F). Furthermore, when coronin 1-deficient macrophages were stimulated with IFN-γ, PKC was readily activated (Fig. 3EF), indicating that PKC activation precedes coronin 1 delocalization.

To understand the contribution of PKC activation to the lysosomal delivery of internalized cargo, macrophages were either left untreated or stimulated for different time periods in the absence or presence of chelerythrine followed by infection with mycobacteria. Activation of macrophages by IFN-γ was sufficient to result in lysosomal transfer of mycobacteria, whereas the presence of the PKC inhibitor chelerytrine prevented mycobacterial delivery to lysosomes (Fig. S4A). Also, direct induction of PKC activity by PMA readily resulted in lysosomal delivery, which was prevented by the inclusion of chelerythrine (Fig. S4B). Thus, macrophage activation by IFN-γ activates PKC which in turn causes the redistribution of cortical coronin 1. As a result, mycobacterial cargo, that is normally retained in non-lysosomal phagosomes is efficiently delivered to lysosomes via macropinocytosis.

We next analyzed whether direct phosphorylation of coronin 1 was responsible for IFN-γ-mediated coronin 1 relocation from the cell cortex to cytoplasmic puncta. The primary sequence of coronin 1 contains several potential PKC consensus sites [19], [39], and analysis of coronin 1 purified from resting and activated macrophages revealed serine phosphorylation but not tyrosine phosphorylation of coronin 1 upon activation while threonines were phosphorylated in both resting and activated cells (Fig. S4C). Subsequent two-dimensional IEF-PAGE revealed the appearance of 4 additional spots in activated, but not resting macrophages, suggesting that interferon-γ activation induces phosphorylation of 4 serines on coronin 1 (Fig. S4D). As expected, the protein kinase C inhibitor chelerythrine blocked activation-induced serine phosphorylation on coronin 1 (Fig. 3G and Fig. S4C). In activated coronin 1-deficient as well as IFN-γ receptor deficient macrophages, none to background levels of coronin 1 or serine phosphorylation was observed (Fig. S4D).

Bioinformatic analysis suggested a high probability for serines 9, 311, 356 and 412 for phosphorylation by PKC (See Material and Methods). To directly analyze the involvement of these serines in coronin 1 relocation, these four residues were mutated to alanine or phosphomimetic glutamate and expressed in coronin 1-deficient macrophages as C-terminal EGFP fusion proteins (Fig. 3H,I). While wild type coronin 1-EGFP localized to the cell cortex in non-activated macrophages, as observed for non-tagged coronin 1 [19], [21], [40] macrophage activation resulted in the relocation of coronin 1-EGFP to cytoplasmic puncta (Fig. 3I). Furthermore, while upon macrophage activation the serine to alanine mutant of coronin 1-EGFP, coronin 1^S-A^-EGFP, failed to relocate to cytoplasmic puncta, the phosphomimetic glutamate mutant of coronin 1-EGFP (coronin 1^S-E^-EGFP) did not localize at the cell cortex but instead localized within cytoplasmic puncta (Fig. 3I). Similarly, upon subcellular fractionation wild type coronin 1-EGFP was predominantly recovered in the pellet fraction, suggesting membrane association, and relocalized to the cytoplasmic fraction (‘supernatant’) upon macrophage activation (see Fig. S4E,F). However, the alanine mutant of coronin 1 remained associated with the pellet fraction in both resting and activated cells, while in contrast, the phosphomimetic mutant was localized to the soluble fraction even in non-activated cells (Fig. S4E,F). Together these data indicate that cytokine-induced macrophage activation results in PKC-mediated coronin 1 phosphorylation on serine 9, 311, 356 and 412 that is a pre-requisite for coronin 1 relocation from the plasma membrane to cytoplasmic puncta.

### Serine Phosphorylation of Coronin 1 Is Necessary and Sufficient for the Induction of Macropinocytosis

The above results suggest that macrophage activation resulted in PKC-mediated coronin 1 phosphorylation concomitant with the induction of macropinocytosis. However, whether or not coronin 1 is directly involved in the induction of macropinocytosis remained unclear. We therefore initiated a series of experiments to analyze whether or not coronin 1 phosphorylation was sufficient for the induction of macropinocytosis, even in the absence of macrophage activation.

**Figure 4:**
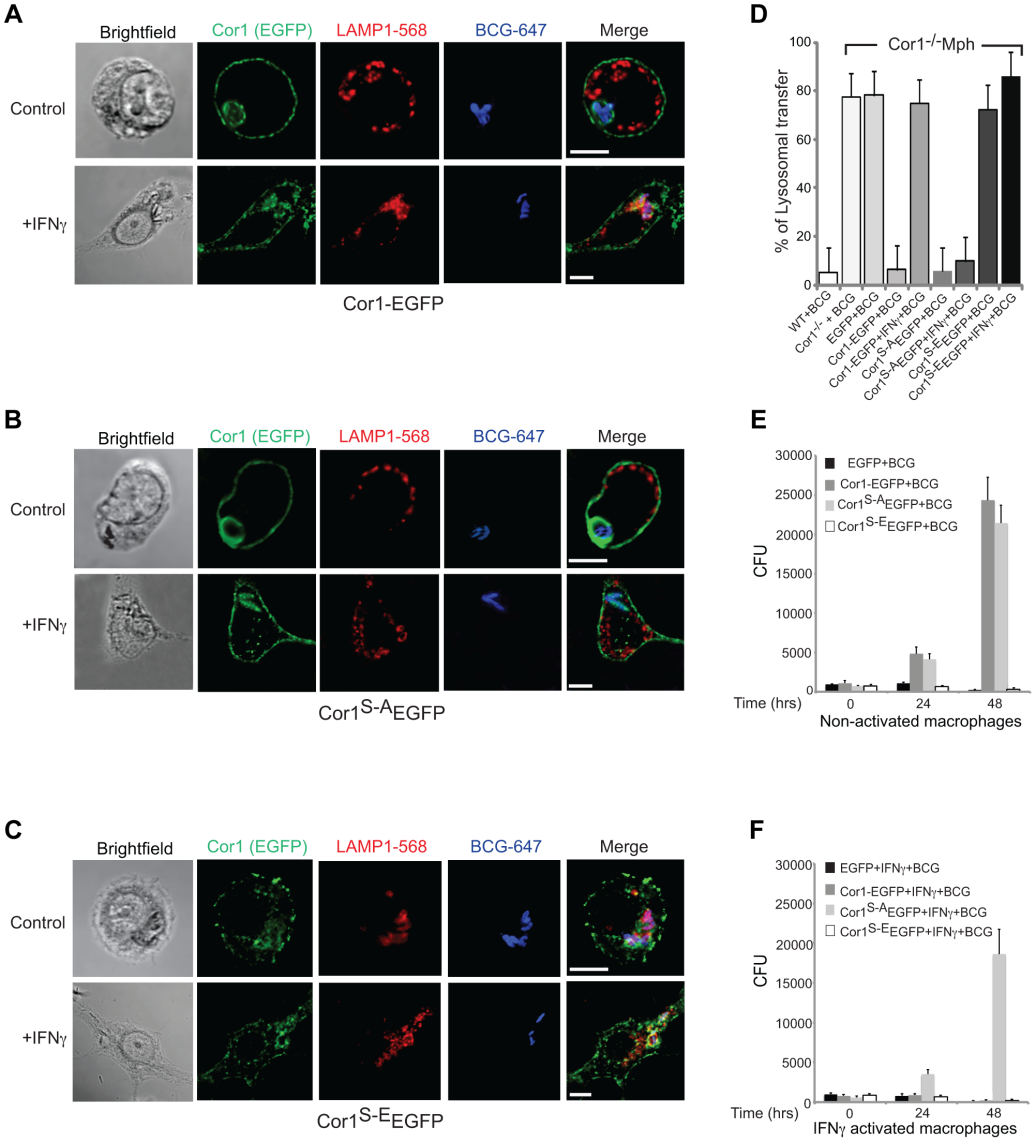
Importance of serine phosphorylated coronin 1 for mycobacterial killing and cargo delivery via macropinocytosis. A–D. Coronin 1-deficient macrophages expressing Cor1-EGFP (A), Cor1^S-A^EGFP (B) or Cor1^S-E^EGFP (C), were left untreated or stimulated with IFN-γ followed by infection with *M. bovis* BCG. Cells were fixed and stained with anti-LAMP1 and anti-mycobacterium antibodies followed by staining with AlexaFluor568- and AlexaFluor647-conjugated secondary antibodies, respectively. Bar: 10 µm. D. Quantitation represents percentage colocalization of bacteria with LAMP1 in cells expressing the indicated constructs (n = 20; three independent experiments). E,F. Survival of *M. bovis* BCG in coronin 1-deficient macrophages expressing EGFP alone, Cor1-EGFP, Cor1^S-A^EGFP and Cor1^S-E^EGFP respectively, either in non-activated(E) or activated (F) macrophages (mean values ± SD from 3 independent experiments).

First, to analyze the consequences of coronin 1 phosphorylation for mycobacterial internalization within macrophages, coronin 1-deficient macrophages expressing the EGFP tagged wild type (Cor1-EGFP), the serine-to-alanine (coronin 1^S-A^-EGFP) mutant or the phosphomimetic mutant (coronin 1^S-E^-EGFP, see Fig. S5A) were infected with mycobacteria for 1 hour, followed by a 3-hour chase and analyzed by confocal microscopy analysis as well as for mycobacterial survival. While in coronin 1-deficient macrophages, as expected [21] [41], mycobacteria were rapidly transferred to lysosomes and killed even without activation (Fig. 4D,E,F), upon expression of wild type coronin 1-EGFP macrophage activation was required to induce lysosomal transfer and killing of internalized mycobacteria (Fig. 4A and panels D–F). However, when IFN-γ-activated coronin 1-deficient macrophages expressing the alanine mutant (coronin 1^S-A^-EGFP) were infected with mycobacteria, lysosomal transfer did not occur (Fig. 4B,D) and the mycobacteria proliferated within macrophages (Fig. 4E,F). This suggest that serine phosphorylation of coronin 1 is essential to relocate mycobacteria to lysosomes upon activation. Conversely, expression of the phosphomimetic coronin 1 mutant (coronin 1^S-E^-EGFP) resulted in all mycobacteria being transferred to lysosomes followed by their elimination, regardless of the macrophage activation state (Fig. 4C,D and panels E,F). These data show that even in the absence of inflammatory stimuli, coronin 1 phosphorylation is sufficient to redirect phagocytic cargo via macropinocytosis to lysosomes.

**Figure 5:**
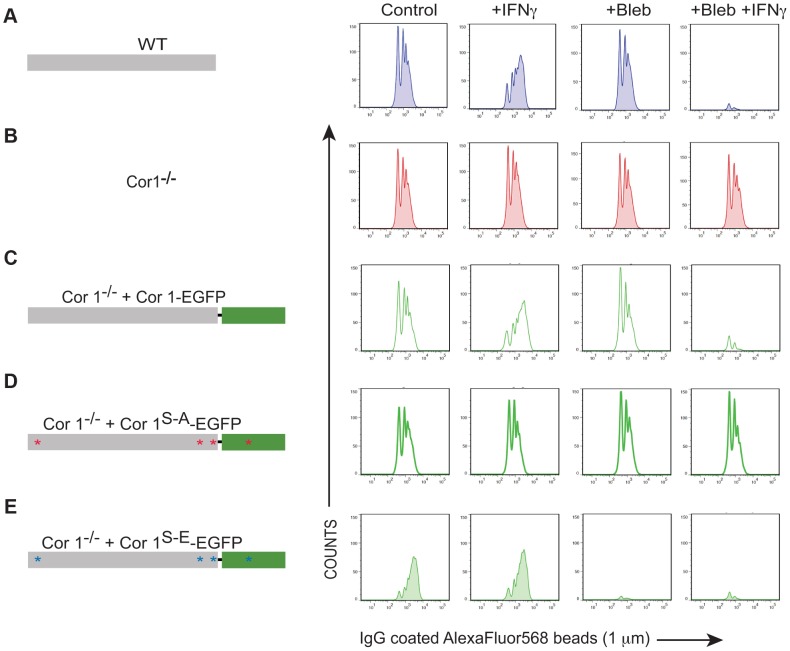
Analysis of IgG-coated bead uptake in cells expressing wild type and serine-phosphorylated mutants of coronin 1. Flowcytometry of IgG-coated AlexaFluor568 beads (1 µM) in wild type (A) or coronin 1-deficient bone marrow-derived macrophages (B) or coronin 1-deficient macrophages transfected with Cor1-EGFP (C), Cor1^S-A^EGFP (D) or Cor1^S-E^EGFP (E) respectively either unstimulated or stimulated with IFN-γ and with or without pre-treatment with blebbistatin. Shown is a representative result out of three experiments.

To analyze whether coronin 1 phosphorylation on serines is a general switch from phagocytosis to macropinocytosis, wild type or coronin 1-deficient macrophages expressing either wild type coronin 1, the serine – alanine coronin 1 mutant or the phosphomimetic coronin 1 mutant as EGFP fusion proteins were incubated with IgG-coated fluorescent beads. As shown in Fig. S5,BC, constitutive macropinocytosis in either resting or activated macrophages was unaltered by transfection of coronin 1 mutants. However, while in wild type cells, as well as in coronin 1-deficient macrophages transfected with wild type coronin 1, macropinocytic uptake of IgG-coated beads was only seen following macrophage activation with interferon-γ (Fig. 5A–C), coronin 1-deficient macrophages alone did not show macropinocytic uptake of beads even upon activation. Notably, in cells expressing the serine-to-alanine (coronin 1^S-A^-EGFP) mutant macropinocytosis did not occur, even upon macrophage activation with interferon-γ (Figure 5D). Moreover, when macrophages expressed the phosphomimetic glutamic acid mutant of coronin 1 (coronin 1^S-E^-EGFP), the IgG-coated beads were internalized through macropinocytosis even in the absence of interferon-γ (Fig. 5E). These results suggest that serine phosphorylation of coronin 1 is the crucial switch from a phagocytic to a macropinocytic uptake mode upon macrophage activation.

### Transient Association of Coronin 1 and Sorting Nexin 5 in Activated Macrophages

**Figure 6:**
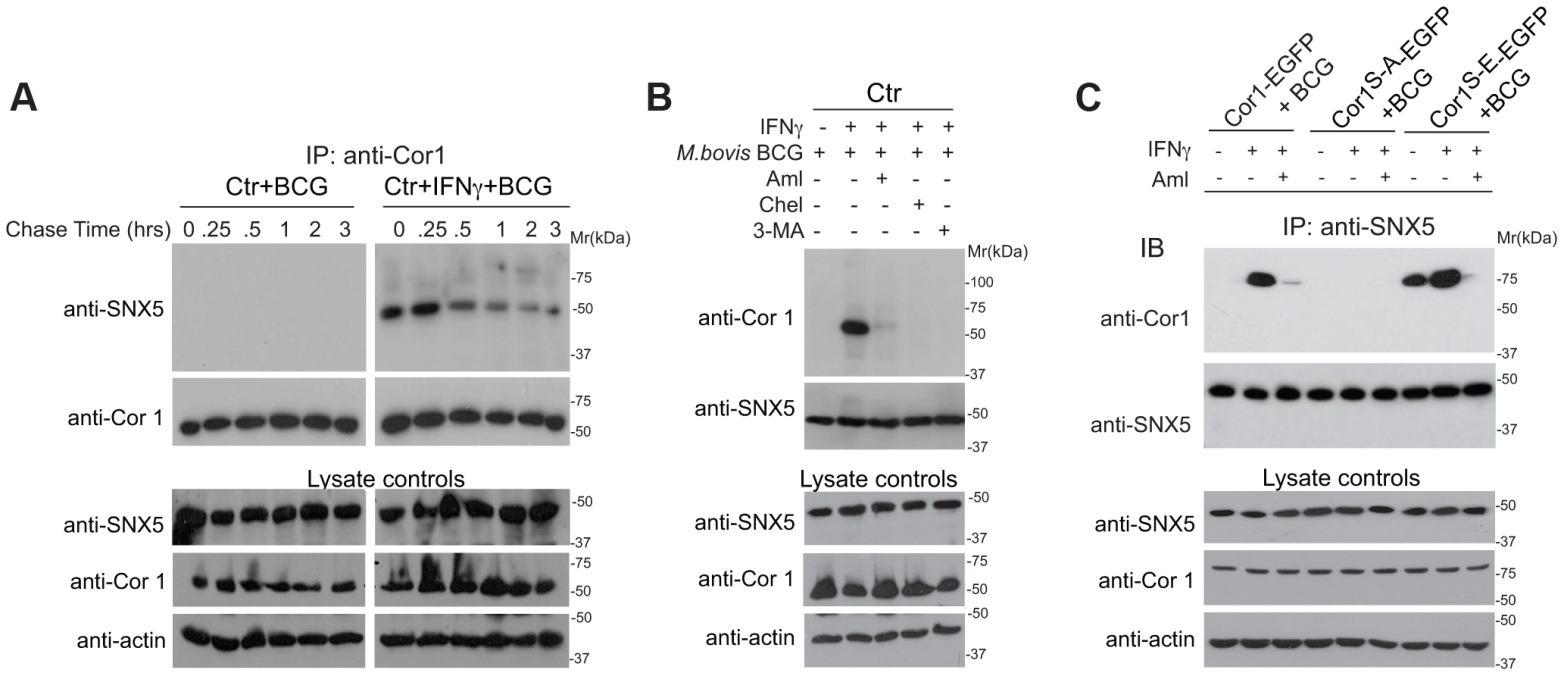
Association of sorting nexin 5 with coronin 1 in IFN-γ activated macrophages. A. Macrophages were left untreated or activated with IFN-γ for 20 hrs, incubated with *M. bovis* BCG for 1 hr followed by the chase times indicated, lysed and immunoprecipitated with anti-coronin 1 antibodies, separated by SDS-PAGE and immunoblotted with anti-SNX5 and anti-coronin 1 antibodies. Separately, lysates were immunoblotted using anti-coronin 1, anti-SNX5 and anti-actin antibodies. B,C. Macrophages from wild type mice (B) or coronin 1-deficient mice transfected with the indicated constructs (C) were left untreated or activated with IFN-γ for 20 hrs, in the absence and presence of the inhibitors indicated and incubated with *M. bovis* BCG for 1 hr followed by a 30 min chase. Cells were lysed and immunoprecipitated with anti-SNX5, separated by SDS-PAGE and immunoblotted with anti-coronin 1 and anti-SNX5 antibodies. Separately, lysates were immunoblotted using anti-SNX5, anti-coronin 1 and anti-actin antibodies.

Macropinocytosis depends on the expression and recruitment of the phosphoinositol-binding protein sorting nexin 5 (SNX5) [42], [43]. Immunoprecipitation of the different sorting nexins from activated and infected macrophages followed by immunoblotting for coronin 1 revealed the specific association of coronin 1 with sorting nexin 5, but not with other sorting nexins (Fig. S6A,B). Coronin 1 was not associated with sorting nexin 5 in resting and infected macrophages (Fig. 6A, left panels and Fig. S6C) and the association between coronin 1 and sorting nexin 5 decreased with increased chase times following mycobacterial infection as analyzed by co-immunoprecipitation and immunofluorescence analysis (Fig. 6A, right panels and Fig. S6D, right panels), suggesting that the association was transient. Furthermore, inclusion of the macropinocytosis inhibitors amiloride or 3-MA as well as the protein kinase C inhibitor chelerythrine prevented association between coronin 1 and sorting nexin 5 (Fig. 6B); Finally, consistent with the importance of serine phosphorylation of coronin 1 for the induction of macropinocytosis upon activation, sorting nexin 5 was not associated nor colocalized with coronin 1^S-A^-EGFP, whereas sorting nexin 5 was associated and colocalized with the phosphomimetic form of coronin 1 (coronin 1^S-E^-EGFP) even in the absence of IFN-γ-mediated activation (Fig. 6C and Fig. S6E,F). These results suggest that during infection, following delocalization from the cell cortex into cytoplasmic puncta upon macrophage activation, serine-phosphorylated coronin 1 is relocalized to nascent macropinosomes in a complex with sorting nexin 5.

### Requirement for Coronin 1 for the Activation of Phosphoinositol-3-kinase Activity upon Macrophage Activation

**Figure 7:**
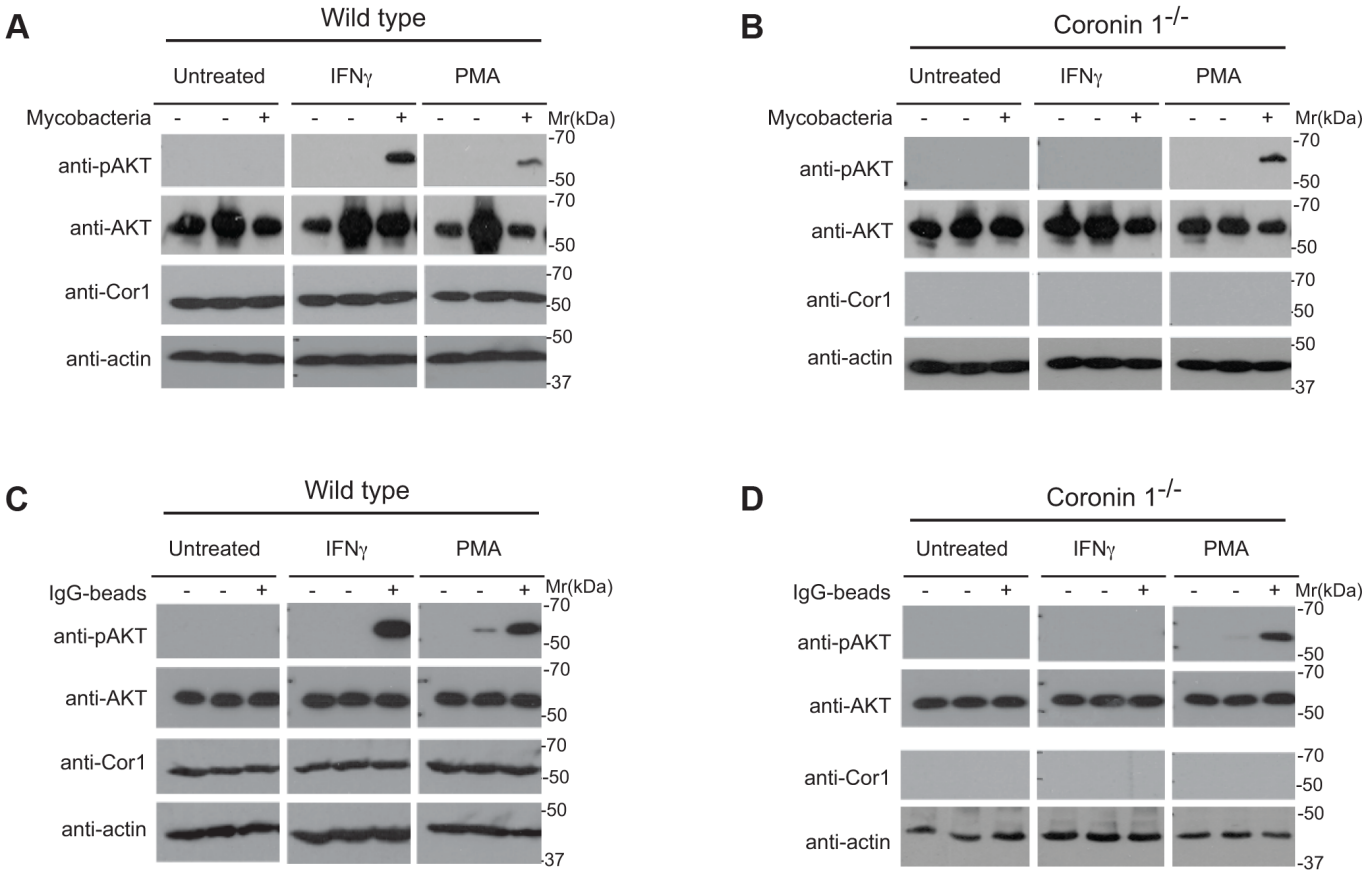
Induction of PI 3 kinase activity in the presence and absence of coronin 1. A,B. Wild type (A) or coronin 1-deficient (B) macrophages were left untreated or activated with IFN-γ (20 hr) or PMA (4 hrs.) followed by incubation with *M. bovis* BCG for 0 (−) or 5 (+) min. Cells were lysed and immunoblotted using anti-phospho AKT (Ser473), anti-panAKT, anti-coronin 1 and anti-actin antibodies. The first lanes in each panel represent lysates from cells to which no bacteria were added. C,D. Wild type (C) or coronin 1-deficient (D) macrophages were left untreated or activated with IFN-γ (20 hr) or PMA (4 hrs.) followed by incubation with IgG-coated beads for 0 (−) or 30 (+) min. Cells were lysed and immunoblotted using anti-phosphoAKT (Ser473), anti-panAKT, anti-coronin 1 and anti-actin antibodies. The first lanes in each panel represent lysates from cells to which no beads were added.

Macropinocytosis is crucially dependent on the activation of the lipid kinase phosphoinositide 3-kinase (PI-3 kinase [17], [33]). Given the relocation of serine-phosphorylated coronin 1 to macropinosomes in association with sorting nexin 5 and the possible association of coronin 1 with PI-3 kinase [44], we asked whether coronin 1 was involved in phosphoinositide 3-kinase activation by monitoring the phosphorylation of Akt/protein kinase B on Ser-473 [45]. Although PI-3 kinase is activated both following phagocytosis as well as macropinocytosis [33], the ruffle formation involved in macropinocytosis is associated with immediate phosphatidylinositol (3,4,5)-trisphosphate (PIP3) generation through rapid PI-3 kinase activity upon addition of cargo, which precedes requirement for PI-3 kinase for cup-closure in both phagocytosis and macropinocytosis [17], [46]. To monitor rapid PI-3 kinase activation, phosphorylation of the downstream substrate AKT on serine 473 was analyzed by immunoblotting of lysates from either resting or activated macrophages to which bacilli or IgG-coated beads had been added. As shown in Figure 7A, incubation of interferon-γ-activated, but not resting macrophages with mycobacteria resulted in substantial AKT phosphorylation at early time points, similar to the activation of AKT by PMA (Fig. 7A). Strikingly, in coronin 1-deficient macrophages, no AKT phosphorylation was detected upon incubation of activated macrophages with mycobacteria, despite similar phosphorylation when incubated with PMA (Fig. 7B). As expected, incubation for longer time points (∼90 mins) resulted in AKT phosphorylation both in resting and IFN-γ-activated macrophages (data not shown). Similar to the coronin 1-dependent PI-3 kinase activation by mycobacteria in interferon-γ-activated macrophages, incubation of IgG-coated beads induced AKT phosphorylation only in wild type, but not coronin 1-deficient macrophages (Fig. 7C,D). These data suggest that coronin 1 is essential for the activation of PI-3 kinase during macropinocytic uptake.

**Figure 8:**
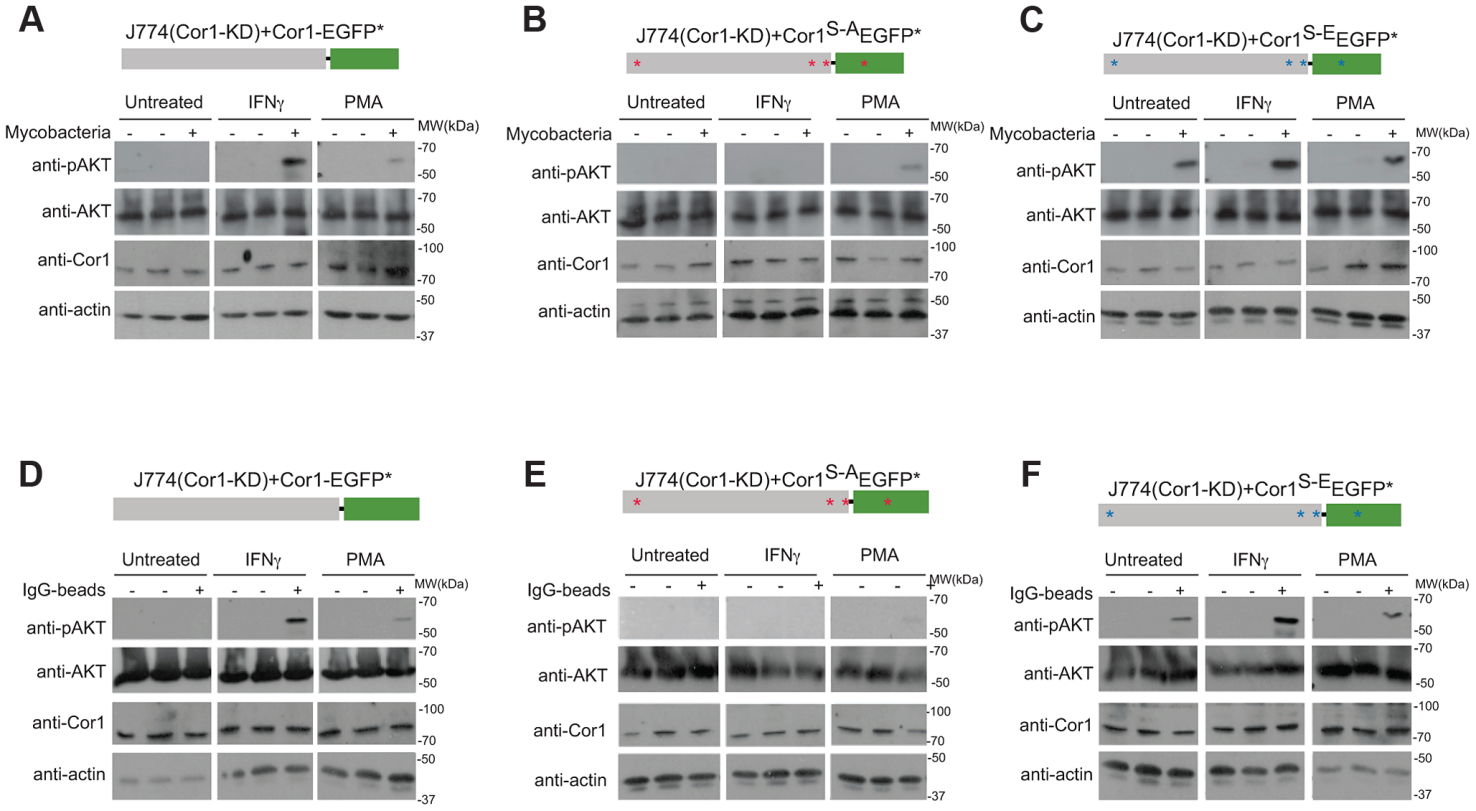
Importance of coronin 1 serine phosphorylation for the induction of phosphatidyl-inositol-3 kinase activity. A–C. J774 macrophages knocked down for coronin 1 were transfected with the indicated constructs and left untreated, activated with IFN-γ (20 hrs.) or PMA (4 hrs.) followed by incubation with mycobacteria for 0 (−) or 30 (+) min. Cells were lysed and immunoblotted using anti-phosphoAKT (Ser473), anti-panAKT, anti-coronin 1 and anti-actin antibodies. The first lanes in each panel represent lysates from cells to which no bacteria were added. D–F. J774 macrophages knocked down for coronin 1 were transfected with the indicated constructs and left untreated, activated with IFN-γ (20 hrs.) or PMA (4 hrs.) followed by incubation with IgG-coated beads mycobacteria for 0 (−) or 30 (+) min. Cells were lysed and immunoblotted using anti-phosphoAKT (Ser473), anti-panAKT, anti-coronin 1 and anti-actin antibodies. The first lanes in each panel represent lysates from cells to which no beads were added.

To analyze the importance of serine phosphorylation on coronin 1 for the activation of PI-3 kinase, J774 macrophages or J774 cells in which coronin 1 expression was knocked down by RNAi ([20], see also Fig. S7) were transfected with either RNAi-resistant wild type, serine-to-alanine or the phosphomimetic form of coronin 1 fused to EGFP, and the resulting cells were incubated with mycobacteria or IgG coated beads in resting and IFN-γ-activated macrophages (Fig. 8 and S7). Expression of wild type coronin 1 in these knock-down cells restored AKT phosphorylation following incubation with either mycobacteria or IgG-coated beads (Fig. 8A,D). Importantly, while expression of the serine-alanine mutant failed to show AKT phosphorylation in either resting or activated cells (Fig. 8B,E), in cells expressing the phosphomimetic coronin 1 mutant, AKT was phosphorylated rapidly even in the absence of interferon-γ (Fig. 8C,F).

Together these results suggest an essential role for serine-phosphorylated coronin 1 in the activation of PI-3 kinase to induce macropinocytosis upon macrophage activation.

## Discussion

During inflammation, immune defense mechanisms must be upregulated to ensure a coordinated response towards the invaded infectious microbes. We here demonstrate that inflammatory stimuli reprogram the macrophage endocytic pathway from phagocytosis to macropinocytosis in a coronin 1-dependent manner. Reprogramming receptor-mediated phagocytosis to macropinocytosis allows macrophages to internalize cargo by bulk flow, rather then being restricted by specific receptor interactions; furthermore, internalizing material through macropinocytosis allows macrophages to efficiently target all incoming microbes to lysosomes for degradation. This may be especially important in the case of pathogens that can survive within non-activated macrophages by resisting phagosome-lysosome fusion, such as *Mycobacterium* spp. Also, although some bacteria can induce macropinocytic entry into non-phagocytes [17], most bacteria enter macrophages via phagocytosis and do not co-opt the macropinocytic pathway. Therefore, the ability of macrophages to switch from phagocytosis to macropinocytosis endows these cells with a mechanism to rapidly eliminate infectious material in lysosomes during an inflammatory challenge.

**Figure 9:**
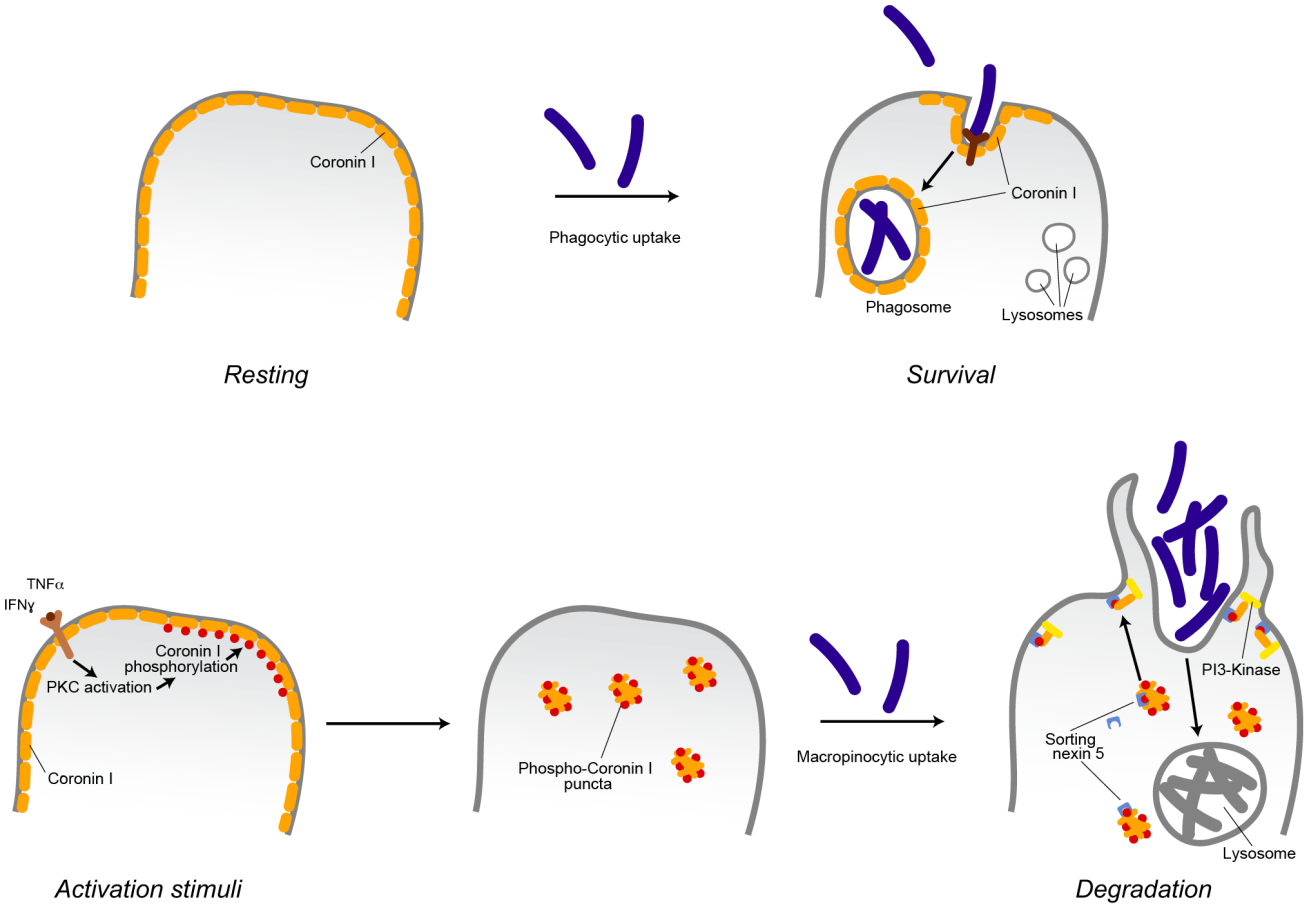
Model describing the induction of coronin 1-mediated macropinocytosis upon macrophage activation. Inflammatory stimuli induce a switch from phagocytosis to macropinocytosis. In resting (non-activated) macrophages, cargo is internalized via receptor-mediated phagocytosis. For the example shown, internalization of pathogenic mycobacteria results in the delivery to phagosomes, where they are protected from lysosomal delivery and killing through recruitment of the host protein coronin 1. Macrophage activation by inflammatory stimuli such as interferon-γ or tumor necrosis factor α results in the activation of protein kinase C that phosphorylates coronin 1 on serine residues, causing a relocalization of phosphorylated coronin 1 from the cell cortex to cytoplasmic puncta. Upon cargo addition, phosphorylated coronin 1 interacts with sorting nexin 5 in order to activate phosphatidylinositol-3 kinase to initiate macropinocytosis at the site of macropinocytic uptake resulting in bulk uptake and lysosomal delivery of the internalized cargo. Whereas constitutive macropinocytosis occurs independent of coronin 1 [21], coronin 1 phosphorylation is essential to re-program phagocytosis to macropinocytosis.

The molecular switch inducing macropinocytosis of cargo upon macrophage activation was revealed to be serine phosphorylation of coronin 1, that was found to directly activate of the lipid kinase phosphatidylinositol 3-kinase, which is required for macropinosome formation, (see also Figure 9). Phosphorylation on coronin 1 occurred on residues 9, 311, 356 and 412 and induced coronin 1 relocation from its location at the cell cortex to cytoplasmic puncta. Upon addition of cargo, coronin 1 assembled with sorting nexin 5 and relocalized to the cell cortex to activate phosphatidylinositol 3-kinase and macropinocytosis.

### Induction of Macropinocytosis upon Macrophage Activation

Importantly, the here demonstrated switch in phagocytic to macropinocytic uptake upon macrophage activation is based on several independent lines of evidence; first, morphological analysis by light and electron microscopy revealed that upon macrophage activation cargo was internalized into spacious vacuoles involving large membrane ruffles, which are hallmarks of macropinocytic as opposed to pseudopod-mediated phagocytic uptake [14], [26]–[29]. Second, upon macrophage activation, internalized cargo strongly colocalized with the early macropinocytic markers SNX1 and SNX 5. Third, we made use of three inhibitors that block macropinocytosis via distinct modes of action; while amiloride blocks macropinocytosis via inhibition of the Na+/H+ exchanger [32], 3-methyl adenosine inhibits macropinocytosis via blocking a specific class of PI-3-kinase and blebbistatin blocks macropinocytosis via the inhibition of nonmuscle myosin II [36], [47] all of which have a crucial role in macropinocytosis. Fourth, analysis by flow cytometry showed that upon macrophage activation, internalization of IgG-coated beads occurred in bulk as opposed to single uptake events in non-activated macrophages.

Induction of macropinocytosis through cytokine-mediated macrophage activation is an exquisite strategy from the host immune defense point-of-view to efficiently eradicate pathogenic material; First, this pathway provides a way for the immune system to clear large amounts of extracellular material [11], [48]; second, since some receptor-mediated phagocytic entry pathways result in silencing of inflammatory responses [12], avoiding phagocytosis altogether and instead taking up cargo via macropinocytosis ensures a complete microbial clearance. Third, there is little to none communication between macropinosomes and conventional endosomes [49], and it is conceivable that this strict separation between macropinosomes and the phagocytic/endocytic pathway may help to ensure a rapid and efficient clearance of the pathogens through lysosomal degradation in activated macrophages, thereby preventing extensive exchange of microbes to more hospitable subcellular organelles such as used by several intracellular pathogens including *Listeria* spp., *Brucella* spp. and *Mycobacterium* spp.

### Coronin 1 and the Induction of Macropinocytosis

An intriguing finding in this study is that the macrophage protein that functions as a switch from phagocytosis to macropinocytosis is coronin 1. Coronin 1 was identified in a search for molecules that allow intracellular survival of mycobacteria that are being internalized through phagocytosis via one of the macrophage phagocytic receptors [1], [18], [19], and subsequent work revealed a role for coronin 1 in promoting Ca^2+^/calcineurin signaling upon mycobacterial infection, but any other role for coronin 1 in macrophages has not been defined [21], [50], [51]. Although other members of the coronin protein family are expressed in macrophages [20], the here described role for coronin 1 in acting as a molecular switch to induce macropinocytosis is clearly a non-redundant function, since in coronin 1-deficient macrophages macropinocytosis was not induced. Why coronin 1 is unable to prevent the delivery of mycobacteria from macropinosomes to lysosomes may lie within the phosphorylation-induced monomerization of coronin 1, which is not anymore capable of protecting the macropinosome residing mycobacteria from lysosomal destruction (SBDG and JP, unpublished).

A function for coronin 1 in the activation of macropinocytosis is furthermore consistent with the association of coronin 1 with cholesterol [24], [52]. Cholesterol is an essential component of the macropinocytic pathway in the absence of which macropinocytosis cannot occur [48], [53]. Interestingly, the cholesterol-staining agent filipin colocalized with coronin 1 within the cytoplasmic puncta; whether cholesterol is required for an efficient phosphorylation of coronin 1, or rather needed for coronin 1-mediated activation of PI-3 kinase during macropinocytic uptake remains to be established.

Whereas the process of phagocytosis is well characterized, the mechanisms involved in macropinocytic entry are only beginning to become elucidated [13], [15], [17], and which proteins exactly are involved in macropinosome formation is still largely unclear [13]. Importantly, constitutive macropinocytosis is unaltered regardless of the activation state of the macrophages and proceeds even in the absence of coronin 1 [21]. Although recent work shows the importance of sorting nexin 5 in macropinocytosis, the precise spatio-temporal pattern of signaling events leading to the induction of macropinocytosis as well as the precise role for sorting nexin 5 in macropinosome formation remains unclear [17], [43]. SNX5 is recruited to the plasma membrane via its phosphoinositide-(PX) binding domain that binds to PI(3,4)P2 [54]. Therefore, the here reported finding that PI-3 kinase activation was dependent on IFN-γ-mediated coronin 1 phosphorylation is consistent with a model in which serine phosphorylated coronin 1 associates with sorting nexin 5 and is targeted to PI(3,4)P2-containing plasma membrane microdomains, after which PI-3 kinase activity is induced to initiate macropinosome formation [55], see also Fig. 9.

Importantly, the coronin 1-mediated reorganization of the endocytic pathway occurred independently from IFN-γ-mediated activation of gene expression [56], since even in the absence of macrophage activation, expression of a serine phosphomimetic coronin 1 mutant was sufficient to induce macropinocytosis. Thus, inflammatory stimuli, besides inducing the expression of a cohort of genes that are directly involved in microbial killing [56]–[61], can also modulate entry pathways in order to efficiently transfer infectious cargo to lysosomal organelles. Many of the IFN-γ-induced genes contribute to an effective immune response, including up regulation of genes important for the induction of autophagy [56]–[62]. Notably, the late macropinosome/autophagosomal marker LC3b was recruited at late times following cargo internalization, at a time point when early macropinocytic markers were not anymore associated with bacteria-containing vacuoles (data not shown), clearly indicating that coronin 1-mediated macropinocytosis precedes autophagy upon macrophage activation [56], [58], [62].

Coronin 1 is emerging as a leukocyte-specific regulator of intracellular signaling processes, and has been shown to promote both the viability of intracellular mycobacteria as well as T lymphocytes via the activation of Ca^2+^-dependent signaling [23]. Interestingly, a rise in intracellular calcium was shown to be required for macropinocytosis to proceed in dendritic cells [63], [64], and it is possible that relocalized coronin 1 is responsible for the Ca^2+^ rise upon induction of macropinocytosis.

In conclusion, the work described here defines coronin 1 phosphorylation as a master switch inducing macropinocytic uptake of cargo upon cytokine activation, thereby coordinating induction of an entry pathway that allows for the macropinocytic engulfment of large amounts of cargo with an up regulation of genes involved in the antibacterial response. It will be interesting to establish whether or not the manipulation of this pathway may be useful in the development of therapies to induce cargo transfer to lysosomes, including the shuttling of pathogenic mycobacteria to lysosomes for rapid elimination.

## Materials and Methods

### Cells

Macrophages were derived from the bone marrow of wild type, coronin 1-deficient, or IFN-γ receptor deficient mice as described [21] unless stated otherwise. All animal experimentation was approved by the veterinary office of the Canton of Basel-Stadt (approved license number 1893) and performed according to local guidelines (Tierschutz-Verordnung, Basel-Stadt) and the Swiss animal protection law (Tierschutz-Gesetz). Macrophages were immortalized with the J2 virus obtained from culture supernatants of NIH-J2-leuk cell line (kind gift from Prof. U. Rapp; [65]), and confirmed to be of the macrophage lineage by staining with F4/80 and CD11b. When stated, J774 wild type or coronin 1 knock down cells as described before [20] were used. All macrophages were grown in DMEM (Sigma; 4.5 g/l glucose), supplemented with 10% heat inactivated FBS (PAA; low endotoxin) and 2 mM L-glutamine (Sigma). *E. coli* (DH5α) was grown in LB. For mycobacterial infections *M. bovis* BCG *(Pasteur strain), M. bovis* BCG-GFP (*Montreal strain*) [19], [66] or *M. marinum* (strain ZF214Cs, a kind gift from Wilbert Bitter, Amsterdam, the Netherlands) was used which were cultered in 7H9 including 10% OADC enrichment and including kanamycin 50 µg/ml in case of *M. bovis* BCG-GFP.

### Reagents

Interferon-γ and tumor necrosis factor-α were from R & D, stocks of 100 µg/ml were prepared in sterile PBS and for activation 1000 U/ml was used. Phorbol myristate acetate (PMA) was from Sigma, stocks of 1 mM were prepared in DMSO and 100 nM was used for activation. Monodansyl cadaverine (MDC) was from Sigma and 100 mM stocks were prepared in DMSO and used at 200 µM, Cytochalasin D (Sigma) stocks were 5 mg/ml in DMSO and 10 µg/ml was used as final concentration. 5-(N-Ethyl-N-isopropyl)amiloride (EIPA; Sigma) stocks were 100 mM in water and 50 µM was used as final concentration. 3-Methyladenine (Sigma) stocks were 1 M in water and 50 mM was used as final concentration. Blebbistatin (Sigma) stocks were 10 mM in DMSO and 150 µM was used as final concentration. Chelerythrine (Sigma) stocks were 10 mM and 10 µM was used as final concentration, while Herbimycin A (Calbiochem) stocks were 100 ug/ml prepared in DMSO and 100 ng/ml was used as final concentration. Amikacin (Sigma) stocks were 100 mg/ml prepared in water and used at 100 µg/ml final concentration. Filipin (Fluka) stocks were 5 mg/ml prepared in methanol and 50 µg/ml was used as final concentration, FM4-64 (Molecular Probes) stocks of 2 mg/ml were prepared in DMSO and 5 µg/ml was used as final concentration.

### Antibodies

Coronin 1 antibodies were either polyclonal rabbit antisera as described before [40] or monoclonal mouse anti-coronin 1 (Abcam). Other antibodies used were: mouse monoclonal anti-actin (Millipore); goat polyclonal (R&D); goat polyclonal (Santa Cruz Biotech) anti-SNX1, anti-SNX2, anti-SNX3, anti-SNX4 and anti-SNX5 as well as rabbit polyclonal anti-SNX5 (Abcam) and rabbit polylonal anti-rab5a (Santa Cruz Biotech); rat monoclonal anti-LAMP-1 clone 1D4B (IgG2a; developed by T. August and obtained from the Developmental Studies Hybridoma Bank at the University of Iowa); rabbit polyclonal anti-GFP (SantaCruz Biotech), mouse monoclonal anti-phosphoserine, anti-phosphothreonine and anti-phosphotyrosine (Cell Signalling) and rabbit polyclonal anti-*Mycobacterium tuberculosis* (SeroTech); rabbit polyclonal anti-panAKT antibody (Abcam) and rabbit polyclonal phosphoAKT (Ser473) antibody (Cell Signalling). All secondary antibodies (Southern Biotech) for western blotting were horse radish peroxide (HRP)-conjugated goat and donkey anti-rabbit, goat anti-mouse or donkey anti-goat. All secondary antibodies for immunofluorescence (Molecular Probes) were AlexaFluor488, 568 or 647-conjugated anti-rabbit, anti-mouse, anti-rat or anti-goat raised in goat or donkey.

### PKH26 Labeling

PKH26 labeling was performed according to the manufacturer's protocol. Briefly, a total of 5×10^8^ number of each of *M. bovis* BCG, *M. marinum* and *E. coli* were transferred to an 1.5 ml Eppendorf tube and washed 3 times with DMEM (without phenol red). PKH26 solution (Sigma) was prepared by diluting 10 µl of PKH26 dye (Sigma) in 1.5 ml of PKH 26 diluent (Sigma) to a final concentration of 4×10^−6^ M. Thereafter 500 µl of diluted PKH26 solution was added to each bacterial suspension in 500 µ l of DMEM and rotated at RT for 30 min. Thereafter the bacteria were washed once with 1 ml of FBS to stop the labeling and to remove excess PKH26, followed by 3 washes in DMEM (without phenol red). The bacteria were resuspended in DMEM (without phenol red) containing 2% FBS. The OD600 was measured and all the bacterial suspensions were brought to a OD600 of 0.1 before adding them to the macrophages.

### Filipin and FM4-64 Staining

Macrophages were seeded on Teflon-coated 10 well slides (BD Falcon) and either kept non-activated or activated. For FM4-64 staining, the dye at final concentration (5 ug/ml in DMEM) along with AlexaFluor 647 conjugate dextran 70,000 was added to the cells, incubated for 30 min followed by fixation in 4% formaldehyde in Hank's Balanced Salt Solution (HBSS) for 10 min at 4°C. Thereafter slides were blocked and stained for coronin 1 with rabbit anti-coronin 1 (1∶1000, 45 min at room temperature) followed by staining with secondary antibodies (anti-rabbit AlexaFluor 488, 30 min at room temperature). Slides were embedded using Pro-Long antifade (Molecular Probes), mounted with coverslips and analyzed using a Zeiss LSM510 Meta confocal laser-scanning microscope. For Filipin staining, incubation with rhodamine-conjugated dextran 70000 was first carried out as indicated above, followed by coronin 1 staining as stated above. Filipin at 250 µg/ml final concentration was prepared in the secondary antibody solution and incubated in the dark along with the secondary antibody solution, followed by embedding and analysis as above.

### Plasmid Constructs and Transfection

Coronin 1 cloned in pEGFP-N1 served as the wild type control for the mutants. pEGFP-N1 was the vector control while site-directed mutagenesis was carried out to mutate serines 9, 311, 356, 412 to alanine and glutamic acid using primers given in Table S1. RNAi-resistant coronin 1 constructs were generated by mutating the region targeted by RNAi (ACTGGACGAGTAGACAAG to ACTGGACG*T*GT*G*GACAAG with the mutated residues in italics) to nucleotides present in the same region of human coronin 1 by site directed mutagenesis using primers indicated in Table S1. The RNAi mutant Cor1-EGFP constructs were denoted with an (*) at the end. Transfection was carried out initially using Amaxa Nucleofector kit V (Lonza; program T-20) or the Neon Transfection system, 100 µl kit (Invitrogen) using the program: 1720 V, 25 sec and 1 pulse. Fluorescent cells were sorted using a FACS Aria III (Becton Dickinson) and either used directly for localization studies or expanded for immunoprecipitation, immunoblotting and flow cytometry studies.

### Mycobacterial Infection in Macrophages

Mycobacterial infection was carried out as described previously [19]. The mycobacterial inoculum was prepared by centrifuging the initial culture in 7H9 at 445*×g* for 5 min to remove all the clumped mycobacteria. Thereafter mycobacteria were pelleted at 2650*×g* for at 30°C in a swing bucket rotor (Eppendorf 5417R), followed by 3 washes in DMEM and finally diluting it to a O.D of 0.1 prior to addition to the cells. Both non-activated or differentially activated macrophages with or without different pre-treatments were seeded on 10-well glass slides (10000 cells for immunofluorescence) or 48 well plates (for colony forming unit enumeration, CFU) and incubated with mycobacteria at OD 0.1 for 1 hr, treated with amikacin (Sigma, 100 µg/ml) in DMEM, washed with DMEM followed by a chase of the times indicated.

### Quantitations

For quantitation of lysosomal transfer, the number of LAMP1 positive mycobacteria containing cells were divided by the total number of cells analyzed and multiplied by 100 to obtain the percentage of lysosome-transferred mycobacteria. For CFU analysis the samples were diluted 1∶10 and plated onto 7H11 agar plates. Thereafter the colonies formed were counted, multiplied by 10, averaged for 3 independent experiments and plotted for each time point. For co-localization with macropinocytic markers, the number of cells exhibiting macropinocytic markers (SNX1 or SNX5) that co-localized with mycobacteria was divided by the total number of infected cells analyzed for a given time point and multiplied with 100 to result in the percentage of macropinosome-localized mycobacteria. Reorganization of coronin 1 following macrophage activation was carried out using the fluorescence images of non-activated and activated macrophages using Fiji [67]. In brief, the entire cell outline was marked and total cellular fluorescence was obtained (F-total). Thereafter, cell-internal cellular fluorescence was measured by outlining the intracellular region apart from the cell cortex (F-internal). F-total divided by F-internal multiplied by 100 allowed to determine the percentage of reorganized coronin 1 while F-internal subtracted from F-total and then divided by F-total followed by multiplication with 100 resulted in the percentage of cortical Coronin 1.

### Scanning Electron Microscopy

Macrophages were grown on glass coverslips in 12 well plates (5×10^4^ cells per well). Cells were incubated with IFN-γ or TNFα for 20 hrs. or PMA for 4 hrs, prior to incubation with mycobacteria. Specific wells were pre-incubated with blebbistatin, amiloride, cytochalasin D or anti-CR3 antibody for 1 hr prior to incubation with bacteria. *M. bovis* BCG-GFP, *M. smegmatis* or *E. coli* at an MOI of 40 was added to the macrophages and incubated at 37°C for 90 min. Cells were immediately washed three times with ice cold PBS followed by fixation with 2.5% glutaraldehyde (EM grade). After fixing, the cells were processed for scanning electron microscopic analysis using the critical point drying technique [68] followed by analysis using a Phillips XL 30 ESEM. In Fig. 1D, mycobacteria were false-colored using Adobe Photoshop CS (version 5.1).

### Flow Cytometry

Bone marrow-derived macrophages were seeded in 6-well plates (1×10^6^ cells/well) and either non-activated or IFN-γ activated in the absence or presence of different inhibitors. Thereafter complement type 3, mouse IgG or mannan -coated AlexaFluor568 conjugated 1 µm beads or rhodamine-coated Dextran 70000 or PKH26 labeled *M. bovis* BCG, *M. marinum* or *E. coli* in DMEM without phenol red containing 2% FBS was added to the cells and incubated for 60 min at 37°C. Subsequently, cells were washed three times with DMEM+2%FBS and collected by flushing in 300 µl DMEM+2%FBS. After incubation, cells were stained with anti-F4/80-FITC and anti-1-A/1-E-Pacific Blue for 20 min on ice followed by washing three times with DMEM+2%FBS. Just before analysis 5 µl of 7AAD-PerCP (Life technologies) was added to the cells. For compensation, unstained or single stained cells were taken. As a control for apoptotic cells control cells incubated with staurosporine for 3 hrs were taken. Cells were analyzed using a Becton-Dickinson FACS Canto II. The cells expressing the EGFP fusion constructs were sorted prior to the experiment and gated in the GFP channel during analysis and non-transfected cells served as controls. Mean fluorescence intensity of bead or bacteria uptake was obtained by multiplying the average fluorescence intensity (Mean) of the internalized cargo with the total number of cells that had internalized cargo (counts) in a fixed time by a fixed number of cells

### Assay for Protein Kinase C Activity

Macrophages, either wild type, coronin 1-deficient or interferon-γ receptor-deficient, were either non-activated or activated with interferon-γ (20 hrs.), TNFα (20 hrs.) or PMA (4 hrs.) in the absence and presence of chelerythrine (1 µM). Cells were lysed with buffer P (20 mM HEPES-NaOH pH 7.4, 25 mM KCl, 1 mM MgCl_2_, 1% NP-40, 0.25% sodium deoxyclolate along with Halt Protease and phosphatase inhibitor (Thermo Scientific)) for 30 min on ice. Subsequently lysates were centrifuged at 16128*× g* for 5 min, at 4°C and the supernatant was diluted 1∶1 with buffer P without detergents and used for activated PKC analysis using a PKC assay kit (Calbiochem). In brief, equal protein amount of cell lysates were incubated in 96 well plates and mixed with radioactive [α^32^P]ATP and the non-phosphorylated PKC substrate RFARKGSLRQKNV. After an incubation for 30 min at 30°C, the phosphorylated substrate was separated from the residual [α^32^P]ATP using P81 phosphocellulose paper and quantitated by using a scintillation counter. As a positive control, activated PKCα (10 ng, Calbiochem) was mixed in the dilution buffer P (see above) and used in the assay.

### Bioinformatic Analysis

The mouse coronin 1 protein sequence was analyzed using the MotifScan program (http://myhits.isb-sib.ch/cgi-bin/motif_scan). In parallel the sequence was also analyzed using ProtScale (http://web.expasy.org/protscale) for residues with lowest hydrophobicity (Kyte and Doolittle) and highest accessibility. Thereafter the residues were confirmed using NetPhosK. Finally residues 9, 311, 356 and 412 were identified as residues putatively phosphorylated by protein kinase C.

### Purification of Coronin 1, One and Two-dimensional Gel Electrophoresis and Immunoblotting and Gel Filtration

Macrophages either non-activated or activated with IFN-γ or PMA and in the absence and presence of Chelerythrine were grown in 15 cm dishes (4 plates for each sample). Thereafter the cells were washed twice with ice-cold PBS, lysed for 15 min on ice with 5 ml of T-X100 lysis buffer per dish (50 mM Tris-HCl, pH 7.5, 137 mM NaCl, 2 mM EDTA, 1 mM PMSF, 10% glycerol, 1% Triton X-100, 0.05% digitonin along with HALT protease and phosphatase inhibitor (GE healthcare)). Lysates were pooled and centrifuged at 1800*×g* for 5 min. at 4°C. The lysate was passed through a 0.45 µm filter and loaded onto an anti-coronin 1 column prepared by crosslinking anti-coronin 1 rabbit antiserum to NHS-coupled sepharose beads (GE healthcare). The column was washed with 100 mM glycine pH 8 followed by elution of the bound coronin 1 with 100 mM glycine pH 3. Fractions were collected (0.5 mL) and immediately neutralized using 1/10 volume of 1 M Tris-HCl, pH 8. Protein concentration was determined using BCA with bovine plasma gamma globulin (BioRad) as a standard and the coronin 1-containing fractions were concentrated followed by buffer exchange using an Amicon centrifuge column (0.5 ml, 10 kDa cutoff). The fractions (100 µl) were mixed with two-dimensional PAGE buffer (GE healthcare) and traces of Bromophenol blue) and separated on 18 cm pH 4–7 immobilized pH gradient (IPG strips GE healthcare) according to the manufacturers protocol and electrophoresed using a Multiphor system II at step increments up to 3500 V in 30 min followed by a run time of 7 hrs. Subsequently, strips were loaded on top of 10% SDS-PAGE gels of 20 cm length and electrophoresed. Immunoblotting was carried out by semi-dry transfer to nitrocellulose membrane (GE healthcare) as described before [19], [69].

### Immunoprecipitation

Immunoprecipitation was carried out upon lysing cells in the following buffer: 20 mM HEPES-NaOH pH 7.4, 50 mM NaCl, 1 mM MgCl_2_, 1 mM EGTA, 0.5 mM PMSF, 0.4% Igepal CA630 (Sigma), 0.3% Na-β-D maltoside (Sigma), 0.2% digitonin (Sigma), 0.1% NP-40 including a protease and phosphatase inhibitor cocktail from Thermo Scientific. The cells were incubated in lysis buffer on ice for 20 min, followed by centrifugation at 4°C for 10 min at 20,000*× g*. Antibodies were coupled to Dynabeads Protein G using dimethyl pimelidate (DMP) according to the manufacturers protocol (Abcam). Antibody-coupled beads were added to these lysates and incubated overnight at 4°C. Thereafter the beads were washed 4–5 times with lysis buffer using a DynaMag (Invitrogen). Bound antigens were either eluted with 100 µl of 100 mM glycine pH 3 followed by neutralization using 1/10th volume of 1 M Tris-HCl pH 8 and were solubilized by boiling 10 min) in sample buffer and loaded on 10% SDS-PAGE, followed by immunoblotting as described above.

### Cell Fractionation

Cultured cells were resuspended in ice cold homogenization buffer (20 mM HEPES, pH 7.9, 10 mM NaCl, 0.5 mM EDTA, 200 mM sucrose, 0.5 mM PMSF and protease and phosphatase inhibitor cocktail (Aprotinin, Bestatin, E-64, Leupeptin, Sodium fluoride, Sodium orthovanadate, Sodium pyrophosphate, b-glycerophosphate, Thermo Scientific) kept on ice for 10 min and then homogenized on ice in a Dounce homogenizer (10–15 strokes). Subsequently, homogenates were centrifuged for 10 min at 4°C at 400*×g*. The pellet was discarded and the supernatant was centrifuged at 18000*×g* for 15 min at 4°C. The resulting pellet served as the plasma membrane fraction, while the supernatant includes the non-plasma membrane fraction [70]. The pellet was solubilized with 1% sodium-β-D-maltoside in homogenization buffer on ice and then suspended in the same buffer, kept on ice again for 15 min followed by centrifugation at 20000*× g* for 10 min at 4°C. Proteins were quantitated from the pellet and the supernatant fractions, separated by SDS-PAGE (10%) followed by immunoblotting as described above.

### Immunofluorescence Analysis

Macrophages were seeded onto Teflon-coated 10 well slides (BD Falcon) followed by the treatments as indicated. Cells were fixed with 4% paraformaldehyde in phosphate buffered saline (PBS) and permeabilized using 0.2% saponin. After blocking with 5% FBS/BSA in phosphate buffered saline, cells were stained with the primary antibodies as indicated (diluted in Dulbecco's PBS containing 5% FBS) followed by incubation with AlexaFluor-conjugated secondary antibodies (diluted in D-PBS containing 5% FBS). Slides were embedded using Pro-Long antifade (Molecular Probes), mounted with coverslips and analyzed using a Zeiss LSM510 Meta confocal laser-scanning microscope. For quantitation, 25 cells were analyzed in three separate experiments and the mean +/− SD is displayed.

### Immunoblotting to Analyze Macropinocytic Uptake in Activated Macrophages

For the preparation of SDS-PAGE samples of mycobacteria-containing cell fractions, macrophages either non-activated or activated with IFN-γ in the absence and presence of different inhibitors were incubated with *M. bovis* BCG-GFP for 1 hr followed by a chase period of 1 hr. Subsequently, cells were lysed using a Triton X-100 buffer (50 mM Tris-HCl, pH 7.5, 137 mM NaCl, 2 mM EDTA, 1 mM PMSF, 10% glycerol, 1% Triton X-100, 0.05% digitonin along with HALT protease and phosphatase inhibitor (GE healthcare)) followed by addition of glass beads equivalent to 200 µl for 500 µl sample and disrupting mycobacteria in the lysates using a mixer mill (type MM 300; Retsch, Germany) as described before [71]. Cell debris and non-lysed cells were removed by centrifugation (10 min at 10,000*×g*) followed by electrophoresis in 10% SDS-PAGE gels. Immunoblotting was carried out using anti-GFP antibody to specifically monitor internalized mycobacteria.

### Immunoblotting for pAKT Analysis

Bone-marrow derived macrophages and J774 macrophages depleted for coronin 1 by siRNA [20] were transfected with RNAi mutants of Cor1-EGFP, namely Cor1-EGFP*, Cor1^S-A^-EGFP* and Cor1^S-E^-EGFP* were either non-activated or activated with IFN-γ for 20 hrs. or PMA for 4 hrs. prior to infection with *M. bovis* BCG for 5 min or with IgG coated beads (1 µm) for 30 mins. Cells were washed with ice-cold HBSS followed by lysis using Triton X-100 buffer containing protease and phosphatase inhibitors. Proteins from the lysates were electrophoresed in 10% SDS-PAGE and immunoblotted using anti-phosphoAKT (Ser473), anti-panAKT, anti-coronin 1 and anti-actin.

## Supporting Information

Figure S1 **Macrophage activation and induction of macropinocytosis.** A. Macrophages were left untreated or activated with IFN-γ (1000 U/ml), TNFα (1000 U/ml) for 20 hr or PMA (100 nM) for 4 hrs followed by infection with *M. bovis* BCG-GFP (green) for 3 hours. Cells were fixed and incubated with anti-LAMP1 antibodies followed by AlexaFluor568-conjugated secondary antibodies. Bar:10 µm. Quantitation represents percentage colocalization of bacteria with LAMP1 (n = 20; three independent experiments). B. Survival of *M. bovis* BCG in non-activated or IFN-γ or TNFα activated macrophages (mean values ± SD from 3 independent experiments). C. Immunolocalization of sorting nexin 1 (SNX1) and sorting nexin 5 (SNX5) as well as coronin 1 in non-activated macrophages. D,E. Quantitation (n = 20) of the degree of colocalization of (A) SNX1 and (B) SNX5 with mycobacteria-containing vacuoles in interferon-γ-activated macrophages. F. Macrophages were either non-activated or activated with interferon-γ in the absence and presence of the endocytosis inhibitors monodansyl cadaverine (200 µM), cytochalasin D (10 µg/ml), amiloride (50 µM) or 3-methyladenine (10 mM), followed by infection with *M. bovis* BCG. Thereafter cells were lysed in a Mixer Mill and immunoblotted using anti-GFP to analyze the extent of internalized *M. bovis* BCG-GFP. G,H. Flowcytometry of IgG-coated AlexaFluor 568 conjugated 1 µm beads (D) or Rhodamine-Dextran 70000 (E) uptake in macrophages either kept untreated (*left panels*) or pre-incubated with blebbistatin (150 µM) (*middle panels*) or cytochalasin D (10 µg/ml) (*right panels*).
(EPS)

Figure S2 **Analysis of** ***E. coli*** **and** ***M. marinum*** **mannan coated bead uptake by flowcytometry.** A,D. Resting or IFN-γ-activated (20 hrs) macrophages were incubated with *M. marinum* (A) or *E. coli* (D) in the absence or presence of blebbistatin (150 µM), amiloride (EIPA, 50 µM), or cytochalasin D (10 µg/ml) respectively upon incubation with mycobacteria followed by analysis using scanning electron microscopy. Bar: 200 nm. B,E. show flow cytometry results. For each condition, 10000 cells were analyzed. Shown is a representative profile out of three independent experiments. C,F depict the mean fluorescence intensity.
(EPS)

Figure S3 **Relocalization of coronin 1 in tumor necrosis factor α and interferon-γ stimulated macrophages.** A. Wild macrophages, activated with TNFα for the indicated time points were fixed and stained with anti-coronin 1 antibodies followed by AlexaFluor488-conjugated secondary antibody. Bar: 10 µm. B,C. Macrophages were activated with IFN-γ followed by rhodamine-conjugated dextran 70,000 uptake (1 mg/ml) (B) or AlexaFluor 647 conjugated dextran 70,000 uptake (1 mg/ml) along with FM4-64 (5 µg/ml)(C) for 30 min at 37°C. Cells were subsequently fixed and stained for coronin 1 alone (C) or co-stained with Filipin (250 µg/ml) (B).
(EPS)

Figure S4 **Phosphorylation of coronin 1 in activated macrophages.** A, B. Macrophages, either left untreated or activated with interferon-γ (A) or PMA (B), in the absence and presence of chelerythrine and herbimycin A (A) or chelerythrine alone (B), were incubated with *M. bovis* BCG for 1 hr followed by a 3 hrs chase. Thereafter the cells were fixed and stained with anti-LAMP1 antibodies followed by AlexaFluor 568 conjugated secondary antibody. Shown is the percentage of lysosome-transferred mycobacteria with SD ± values from three independent experiments, (n = 25) were plotted. C. SDS-PAGE analysis of coronin 1 purified from non-activated, IFN-γ or TNFα activated macrophages in the absence and presence of chelerythrine. Samples were loaded in 4 sets, electrophoresed, transferred to nitrocellulose and immunoblotted with anti-coronin 1, anti-phosphotyrosine, anti-phosphoserine and anti-phosphothreonine antibodies. D. Coronin 1 purified from interferon-γ receptor-deficient or coronin 1-deficient macrophages after activation with interferon-γ was subjected to 2D IEF/SDS-PAGE gel electrophoresis followed by immunoblotting with anti-coronin 1 and anti-phosphoserine antibodies successively. E. Coronin 1-deficient macrophages transfected with the indicated constructs (EGFP, Cor1-EGFP, Cor1^S-A^-EGFP and Cor1^S-E^-EGFP) were left untreated or activated with interferon-γ followed by lysis and fractionation of pellet (P) and supernatant (S). The fractions were separated by SDS-PAGE and immunoblotted using anti-GFP, anti-coronin 1 and anti-actin antibodies. F. Quantitation of the immunoblot shown in panel F and representing pellet versus soluble fraction of coronin 1 in different transfectants, upon IFN-γ stimulation.
(EPS)

Figure S5 **Internalization of Rhodamine-conjugated Dextran70000 in macrophages expressing wild type and serine mutants of coronin 1.** A. Coronin 1-deficient macrophages were transfected with cDNA encoding wild type Cor1-EGFP, alanine mutant Cor1^S-A^-EGFP or the phosphomimetic glutamic acid mutant Cor1^S-E^-EGFP, lysed and coronin 1 expression analyzed by SDS-PAGE and immunoblotting for coronin 1. BMM: bone marrow-derived macrophages from wild type mice. B. Flowcytometric analysis of rhodamine conjugated dextran 70,000 uptake in wild type (upper panels) or coronin 1-deficient macrophages (lower panels) either non-activated (left panels) or activated with IFN-γ (middle panels) or TNFα (right panels) and in the absence and presence of EIPA. C. For the analysis of Rhodamine internalization, cells as in A were either left unstimulated or stimulated with interferon-γ with or without pretreatment with blebbistatin followed by incubation with Rhodamine-Dextran 70000 (10 µg/ml, 60 min) and analyzed by flowcytometry.
(EPS)

Figure S6 **Immunoprecipitation of sorting nexin isoforms from interferon-γ activated macrophages upon mycobacterial infection.** A,B. Macrophages were stimulated with interferon-γ for 20 hrs and incubated with *M. bovis* BCG for 1 hr. followed by a 30 min chase. Subsequently, cells were lysed and immunoprecipitated using antibodies against the indicated isoforms of sorting nexins or controls (anti-Rab5a), followed by SDS-PAGE and immunoblotting using anti-coronin 1 antibodies (A) or antibodies against sorting nexins (B). C. Resting macrophages were infected with *M. bovis* BCG for 60 min followed by a 30 min chase, fixed, and stained for coronin 1, sorting nexin 5 and mycobacteria. D. Macrophages were activated with interferon-γ for 20 hours and infected with *M. bovis* BCG for 30 or 60 min (left panels), washed, and chased for an additional 30 or 60 min (right panels) followed by fixation and immunofluorescence for coronin 1, sorting nexin 5 and mycobacteria. Bar: 10 µm. E,F. Coronin 1-deficient macrophages expressing Cor1^S-A^EGFP (E) or Cor1^S-E^EGFP were left untreated or activated as indicated followed by infection with mycobacteria for 60 min followed by a 30 min chase. Cells were fixed and stained for coronin 1, sorting nexin 5 and mycobacteria. Bar: 10 µm.
(PDF)

Figure S7 **Analysis of PI-3-kinase activity upon mycobacterial infection in J774 wild type and coronin 1-knock down macrophages.** A,B. J774 wild type (A) or coronin 1 knock down (B) J774 macrophages were left untreated or activated with IFN-γ (20 hrs) or PMA (4 hrs.) .) followed by incubation with *M. bovis* BCG for 0 (−) or 30 (+) min. Cells were lysed, and proteins analyzed by SDS-PAGE followed by immunoblotting using anti-phosphoAKT (Ser473), anti-panAKT, anti-coronin 1 and anti-actin antibodies. The first lanes in each panel represent lysates from cells to which no bacteria were added. C. Coronin 1 knockdown J774 macrophages were either non-transfected or transfected with Cor1-HA*, Cor1-EGFP*, Cor1^S-A^-EGFP* and Cor1^S-E^-EGFP*, lysed and proteins were separated by SDS-PAGE followed by immunoblotting with anti-coronin 1 antibodies.
(EPS)

Movie S1 **Time-lapse microscopy of non-activated, coronin 1-EGFP expressing macrophages.**
(MOV)

Movie S2 **Time-lapse microscopy of coronin 1-EGFP expressing macrophages activated with IFN-γ.**
(MOV)

Movie S3 **Time-lapse microscopy of coronin 1-EGFP expressing macrophages activated with PMA.**
(MOV)

Table S1 **Primers for site directed mutagenesis of Protein kinase C phosphorylated serines in coronin 1 and shRNA mutants of the coronin1-EGFP mutants.**
(PDF)
