# Correction: Inflammatory Stimuli Reprogram Macrophage Phagocytosis to Macropinocytosis for the Rapid Elimination of Pathogens

**DOI:** 10.1371/journal.ppat.1004127

**Published:** 2014-04-16

**Authors:** The PLOS Pathogens Staff

There is an error in Figure 4. The lower left panel of Figure 4C (Brightfield +IFNγ) was rotated relative to the fluorescent panels. The corrected version of Figure 4 can be seen here.

**Figure 4:**
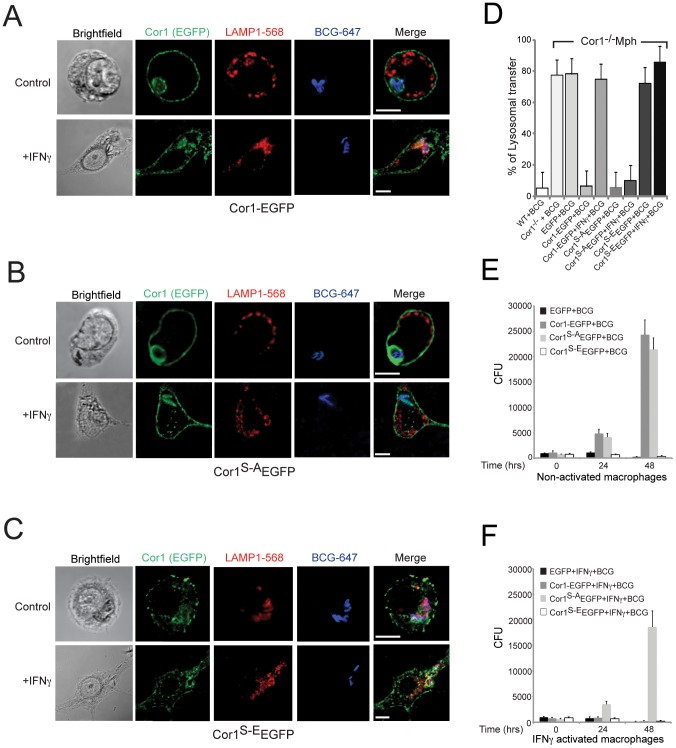
Importance of serine phosphorylated coronin 1 for mycobacterial killing and cargo delivery via macropinocytosis. A–D. Coronin 1-deficient macrophages expressing Cor1-EGFP (A), Cor1^S-A^EGFP (B) or Cor1^S-E^EGFP (C), were left untreated or stimulated with IFN-γ followed by infection with *M. bovis* BCG. Cells were fixed and stained with anti-LAMP1 and anti-mycobacterium antibodies followed by staining with AlexaFluor568- and AlexaFluor647-conjugated secondary antibodies, respectively. Bar: 10 µm. D. Quantitation represents percentage colocalization of bacteria with LAMP1 in cells expressing the indicated constructs (n  =  20; three independent experiments). E,F. Survival of *M. bovis* BCG in coronin 1-deficient macrophages expressing EGFP alone, Cor1-EGFP, Cor1^S-A^EGFP and Cor1^S-E^EGFP respectively, either in non-activated(E) or activated (F) macrophages (mean values ± SD from 3 independent experiments).
