# Supplementary figures and images for: Inflammatory Stimuli Reprogram Macrophage Phagocytosis to Macropinocytosis for the Rapid Elimination of Pathogens
Source: PLoS Pathog. 2014 Jan 30;10(1):e1003879. doi: 10.1371/journal.ppat.1003879 (PMC3907376; doi:10.1371/journal.ppat.1003879)

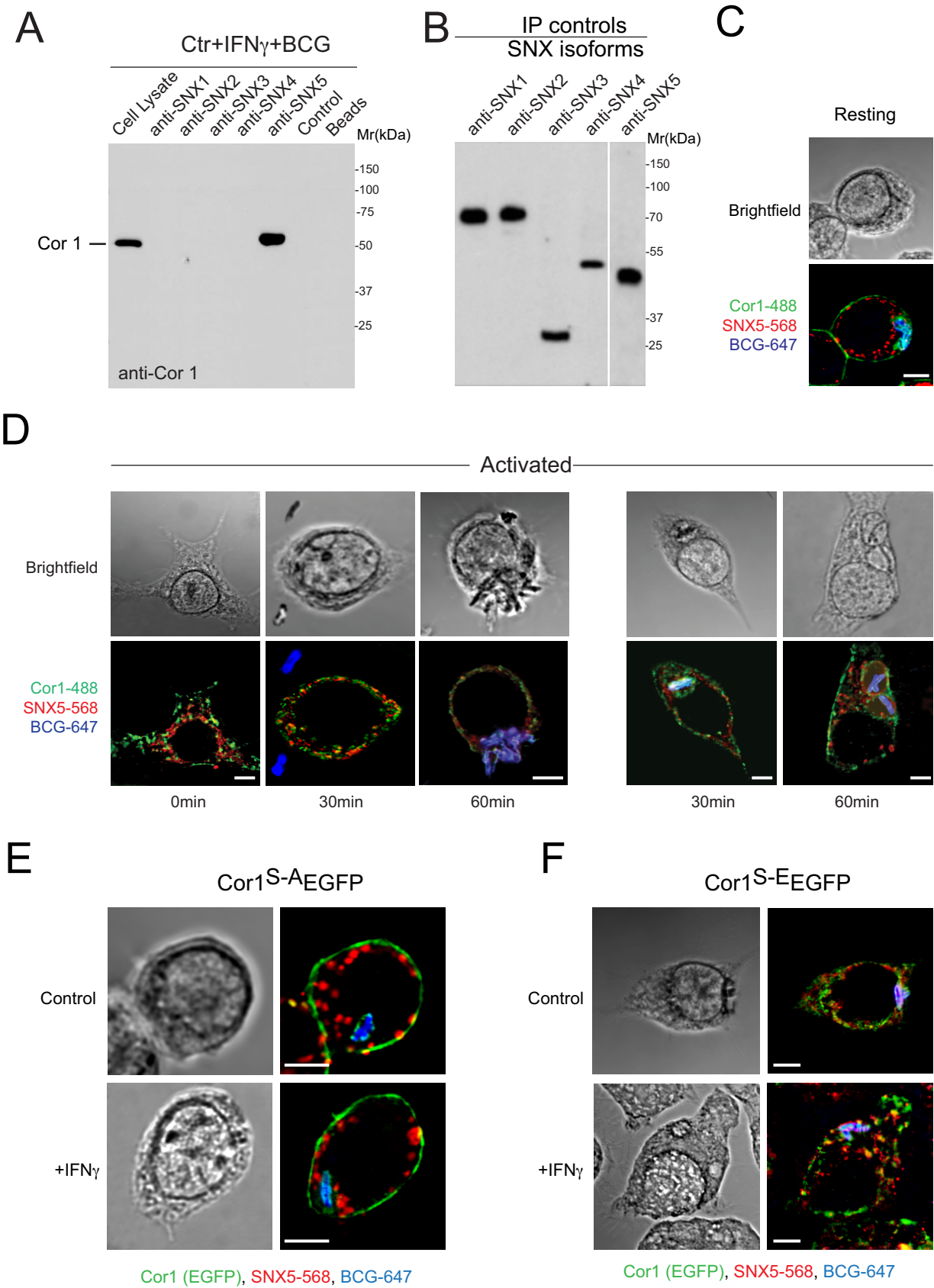

Supplement: Figure S6 — Immunoprecipitation of sorting nexin isoforms from interferon-γ activated macrophages upon mycobacterial infection. A,B. Macrophages were stimulated with interferon-γ for 20 hrs and incubated with M. bovis BCG for 1 hr. followed by a 30 min chase. Subsequently, cells were lysed and immunoprecipitated using antibodies against the indicated isoforms of sorting nexins or controls (anti-Rab5a), followed by SDS-PAGE and immunoblotting using anti-coronin 1 antibodies (A) or antibodies against sorting nexins (B). C. Resting macrophages were infected with M. bovis BCG for 60 min followed by a 30 min chase, fixed, and stained for coronin 1, sorting nexin 5 and mycobacteria. D. Macrophages were activated with interferon-γ for 20 hours and infected with M. bovis BCG for 30 or 60 min (left panels), washed, and chased for an additional 30 or 60 min (right panels) followed by fixation and immunofluorescence for coronin 1, sorting nexin 5 and mycobacteria. Bar: 10 µm. E,F. Coronin 1-deficient macrophages expressing Cor1S-AEGFP (E) or Cor1S-EEGFP were left untreated or activated as indicated followed by infection with mycobacteria for 60 min followed by a 30 min chase. Cells were fixed and stained for coronin 1, sorting nexin 5 and mycobacteria. Bar: 10 µm. (PDF) [file ppat.1003879.s006.pdf]
