# Supplementary material for: Inflammatory Stimuli Reprogram Macrophage Phagocytosis to Macropinocytosis for the Rapid Elimination of Pathogens
Source: PLoS Pathog. 2014 Jan 30;10(1):e1003879. doi: 10.1371/journal.ppat.1003879 (PMC3907376; doi:10.1371/journal.ppat.1003879)
Supplement: Table S1 — Primers for site directed mutagenesis of Protein kinase C phosphorylated serines in coronin 1 and shRNA mutants of the coronin1-EGFP mutants. (PDF) [file ppat.1003879.s011.pdf]

**Table S1**

| Primer | Strand | Sequence |
|---|---|---|
| **S9A** | sense | GGTGGTTCGCTCCGCCAAATTCCGCCACG |
| | antisense | CGTGGCGGAATTTGGCGGAGCGAACCACC |
| **S9E** | sense | GGTGGTTCGCTCCGAGAAATTCCGCCACG |
| | antisense | CGTGGCGGAATTTCTCGGAGCGAACCACC |
| **S311A** | sense | GCATTATCTTTCCATGTTCGCTTCCAAGGAGTCTC |
| | antisense | GAGACTCCTTGGAAGCGAACATGGAAAGATAATGC |
| **S311E** | sense | GCATTATCTTTCCATGTTCGAGTCCAAGGAGTCTC |
| | antisense | GAGACTCCTTGGACTCGAACATGGAAAGATAATGC |
| **S356A** | sense | GACAGTGCCTAGAAAGGCGGACCTGTTCCAG |
| | antisense | CTGGAACAGGTCCGCCTTTCTAGGCACTGTC |
| **S356E** | sense | GACAGTGCCTAGAAAGGAGGACCTGTTCCAG |
| | antisense | CTGGAACAGGTCCTCCTTTCTAGGCACTGTC |
| **S412A** | sense | CAGGGGCCTGGACGCCGCTCGCAGAAGAGC |
| | antisense | GCTCTTCTGCGAGCGGCGTCCAGGCCCCTG |
| **S412E** | sense | CAGGGGCCTGGACGAGGCTCGCAGAAGAGC |
| | antisense | GCTCTTCTGCGAGCCTCGTCCAGGCCCCTG |
| **mutRNAi** | sense | CCCCTAGGCAAGACTGGACGTGTGGACAAGAACGTGCCC |
| | antisense | GGGCACGTTCTTGTCCACACGTCCAGTCTTGCCTAGGGG |

Table S1: Primers for site directed mutagenesis of Protein kinase C phosphorylated serines in coronin 1 and shRNA mutants of the coronin1-EGFP mutants.
